# Embedding Anatomical or Functional Knowledge in Whole-Brain Multiple Kernel Learning Models

**DOI:** 10.1007/s12021-017-9347-8

**Published:** 2018-01-03

**Authors:** Jessica Schrouff, J. M. Monteiro, L. Portugal, M. J. Rosa, C. Phillips, J. Mourão-Miranda

**Affiliations:** 1 Laboratory of Behavioral and Cognitive Neuroscience, Stanford University, Stanford, CA USA; 2 Centre for Medical Image Computing, Department of Computer Science, University College London, London, UK; 3 GIGA Research, University of Liège, Liège, Belgium; 4 Department of Physiology and Pharmacology, Federal Fluminense University, Niterói, RJ Brazil; 5 Max Planck UCL Centre for Computational Psychiatry and Ageing Research, University College London, UK; 6 Department of Electrical Engineering and Computer Science, University of Liège, Liège, Belgium

**Keywords:** Machine learning, Multiple Kernel Learning, Neuroimaging, MATLAB software, Model interpretation, Anatomically defined regions

## Abstract

Pattern recognition models have been increasingly applied to neuroimaging data over the last two decades. These applications have ranged from cognitive neuroscience to clinical problems. A common limitation of these approaches is that they do not incorporate previous knowledge about the brain structure and function into the models. Previous knowledge can be embedded into pattern recognition models by imposing a grouping structure based on anatomically or functionally defined brain regions. In this work, we present a novel approach that uses group sparsity to model the whole brain multivariate pattern as a combination of regional patterns. More specifically, we use a sparse version of Multiple Kernel Learning (MKL) to simultaneously learn the contribution of each brain region, previously defined by an atlas, to the decision function. Our application of MKL provides two beneficial features: (1) it can lead to improved overall generalisation performance when the grouping structure imposed by the atlas is consistent with the data; (2) it can identify a subset of relevant brain regions for the predictive model. In order to investigate the effect of the grouping in the proposed MKL approach we compared the results of three different atlases using three different datasets. The method has been implemented in the new version of the open-source Pattern Recognition for Neuroimaging Toolbox (PRoNTo).

## Introduction

During the last years there has been a substantial increase in the application of machine learning models to analyse neuroimaging data (please see Pereira et al. 2009 and Haynes 2015 for overviews). In cognitive neuroscience, applications of these models -also known as brain decoding or mind reading- aim at associating a particular cognitive, behavioural or perceptual state to specific patterns of brain activity. In the context of clinical neuroscience, machine learning analyses usually focus on predicting a group membership (e.g. patients vs. healthy subjects) from patterns of brain activation/anatomy over a set of voxels. Due to their multivariate properties, these approaches can achieve relatively greater sensitivity and are therefore able to detect subtle and spatially distributed effects. Recent applications of machine learning models to neuroimaging data include predicting, from individual brain activity, the patterns of perceived objects (Haynes and Rees 2005; Ramirez et al. 2014), mental states related to memory retrieval (Polyn et al. 2005) and consolidation (Tambini and Davachi 2013), hidden intentions (Haynes et al. 2007) and semi-constrained brain activity (Schrouff et al. 2012). These techniques also showed promising results in clinical applications (see e.g. Klöppel et al. 2012), providing potential means of computer-aided diagnostic tools for Alzheimer’s disease (Klöppel et al. 2008), Parkinson’s disease (e.g. Orrù et al. 2012; Garraux et al. 2013) or depression (Fu et al. 2008). Accordingly, various software packages have been implemented to ease the application of machine learning techniques to neuroimaging data. To cite a few: The Decoding Toolbox (Hebart et al. 2015), MVPA toolbox, PyMVPA (Hanke et al. 2009a, b), Nilearn (Abraham et al. 2014), Representational Similarity Analysis (Kriegeskorte et al. 2008), CoSMoMVPA (Oosterhof et al. 2016), Searchmight (Pereira and Botvinick 2011), 3Dsvm (LaConte et al. 2005), Probid, Mania (Grotegerd et al. 2014), PETRA or our own work PRoNTo (Schrouff et al. 2013a).

When applying machine learning predictive models to whole brain neuroimaging data a researcher often wants to be able to answer two questions: (1) Which brain regions are informative for the prediction? (2) Why are these regions informative? Considering the first question, although linear models generate weights for each voxel, the model predictions are based on the whole pattern and therefore one cannot arbitrarily threshold the weights to identify a set of informative features (or voxels). Indeed, if one were to threshold a weight map (e.g. by removing voxels/regions with low contribution), the result would be a new predictive function that has not been evaluated. In order to identify which features have predictive information one can use feature selection approaches or sparse models. One limitation of these approaches is that often they do not take into account our previous knowledge about the brain. We know that the brain is organised in regions and the signal within these regions are expected to vary smoothly. One way to incorporate this knowledge into the models is to use structured or group sparsity. A number of studies have shown the benefits of using structured sparse approaches in neuroimaging applications (e.g. Baldassarre et al. 2012, Grosenick et al. 2011). However, these models are computationally expensive and it is difficult to design a structured sparsity that incorporates all characteristics of the neuroimaging data. An alternative way to incorporate knowledge about the data into the models is to use group sparsity. For example, there is evidence that group sparse regularization (i.e. group lasso) can improve recovery of the model’s coefficients/weights in comparison with the lasso when the grouping structure is consistent with the data (Huang and Zhang 2010). Here, we used anatomical/functional information to define the grouping structure and a sparse version of Multiple Kernel Learning (MKL) to simultaneously learn the contribution of each brain region to the predictive model.

The question of why a set of regions carries predictive information is more difficult to answer and has been previously discussed in the literature (e.g. Haufe et al. 2014; Weichwald et al. 2015; Kia et al. 2016). Basically, weights of linear predictive models show the relative contribution of the features for prediction, but do not disentangle potential causes for the contribution. For example, as shown by Haufe and collaborators (Haufe et al. 2014), a feature might have a high weight (or a high contribution) due to an association with the labels or a high weight to cancel correlated noise between the features. Therefore, we argue that additional analysis needs to be done (e.g. univariate statistical tests) to understand why a specific feature (or region) has a high contribution to a predictive model.

In this work, we propose an approach that is able to select a subset of informative regions for prediction based on an anatomical/functional atlas, thereby addressing the first question. However, we do not attempt to address the second question, as we believe multivariate predictive models cannot provide a clear answer to why a specific feature/region has a high contribution to the model (Weichwald et al. 2015). In the present work, we will refer to the ‘interpretability’ of a predictive model as its ability to identify a subset of informative features/regions.

### Related Approaches

Different solutions have been proposed to identify which features contribute to the model’s prediction^1^: Kriegeskorte et al. (2006) proposed a locally multivariate approach, known as “searchlight”, whereby only one voxel and its direct neighbours (within a sphere which radius is defined a priori) are selected to build the machine learning model. This operation is then repeated for all voxels, leading to a map of performance (e.g. accuracy for classification and mean squared error, MSE, for regression). Based on the significance of model performance in each sphere, the resulting maps can be thresholded. While this approach can provide insights on which regions in the brain have a local informative pattern, it presents the disadvantage of considering each sphere independently. The brain is therefore not considered as a whole anymore, but as a collection of partially overlapping spheres, which reduces the multivariate power of machine learning models by focusing only on local patterns. The interested reader can refer to Etzel et al. 2013 for a discussion on the promise, pitfalls and potential of this technique.

Another approach that has been used to threshold weight maps is to perform a permutation test at each voxel to generate a map of *p*-values (e.g. Mourão-Miranda et al. 2005, Klöppel et al. 2008, Marquand et al. 2012, 2014). In this case the labels of the training data are randomly shuffled *p* times and the model is trained using the shuffled labels to generate a null distribution of the models’ weight for each voxel. The voxels with a statistically high contribution (positive or negative) to the model compared to its null distribution can then be highlighted. The resulting statistical maps can be thresholded, using the p-values obtained for each voxel. The correction for multiple comparisons should be performed with care, as detailed in (Gaonkar and Davatzikos 2012). In addition, this approach is computationally expensive.

Some authors have proposed the use of sparse models, like LASSO (Tibshirani 1996) or Elastic-net (Zou and Hastie 2005), as they are able to estimate solutions for which only few voxels are considered relevant. Structured sparse models, such as sparse Total Variation (TV, Baldassarre et al. 2012) and Graph Laplacian Elastic Net (GraphNET, Grosenick et al. 2011), allow incorporation of domain knowledge through additional spatial and temporal constraints and carry the promise of being more interpretable than non-structured sparse methods, such as LASSO or Elastic Net methods. A drawback of the sparse models is that the solution is highly dependent on the way the prior or regularization term is specified. Often models with different regularization terms (e.g. LASSO, Elastic-net, Total Variation) achieve similar accuracies for different solutions (Baldassarre et al. 2012). In this sense, some authors have argued that the quality of spatial patterns extracted from sparse models cannot be assessed purely by focusing on prediction accuracy (Rasmussen et al. 2012).

Feature selection based on stability theory (Meinshausen and Bühlmann 2010) has also been proposed as a mapping approach by identifying a subset of stable features that are relevant to the predictive model (Rondina et al. 2014). This approach relies on the idea of choosing relevant features that are stable under data perturbation. Data are perturbed by iteratively sub-sampling both features and examples. For each perturbation, a sparse method (e.g. LASSO) is applied to a sub-sample of the data. After a large number of iterations, all features that were selected in a large fraction of the perturbations are selected. Although this approach has the potential to identify reliable relevant features for the predictive models, it does not account for prior knowledge about brain anatomy neither for the spatial correlation among the voxels.

Another approach to tackle the interpretability of machine learning models is to use previous knowledge about brain anatomy to segment the whole brain multivariate pattern into regional patterns. This strategy was used in (Schrouff et al. 2013b): the authors proposed local averages of the model weights according to regions defined by the Automated Anatomical Labelling (AAL, Tzourio-Mazoyer et al. 2002) atlas. Regions were then sorted according to their proportional contribution to the weight vector or decision function, thereby providing a ranking of the regions. Even though the results of this study showed that regions ranked in the top 10 (arbitrarily fixed threshold) were in line with previous univariate studies, this approach does not solve the issue of thresholding since for non-sparse machine learning models^2^ (e.g. Support Vector Machines, Kernel Ridge Regression) all brain regions considered will have some contribution to the model’s predictions. Investigating regional contribution through post-hoc summarization was also performed in Hanke et al. 2009a. In their work, the authors matched probabilistic weight maps with anatomical information to derive a ‘specificity’ measure for each region of interest. This approach however suffers from the same limitation, i.e. regions with low sensitivity are part of the decision function and cannot be pruned.

Multiple Kernel Learning (MKL, Bach et al. 2004) approaches have been previously applied in the context of neuroimaging to e.g. perform multi-modal diagnosis of Alzheimer disorders (Hinrichs et al. 2011; Zhang et al. 2011), attention deficit hyperactivity disorder (ADHD) children (Dai et al. 2012), predict cognitive decline in older adults (Filipovych et al. 2011) and discriminate three Parkinsonian neurological disorders (Filippone et al. 2012). In (Filippone et al. 2012), each kernel corresponded to either an image modality or an anatomically labelled region. The authors used the kernel weights to analyze the relative informativeness of different image modalities and brain regions. However, the considered algorithm was not sparse in the kernel combination, making it difficult to determine a subset of regions with highest contribution to the model. Our work differs from Filippone et al. 2012 as we use MKL as an exploratory approach to find a (sparse) subset of informative regions for a predictive model, considering all brain regions a priori defined by a whole brain template.

### Proposed Approach

The proposed framework combines anatomical/functional parcellations of the brain, MKL and sparsity. More specifically, we use a sparse version of the MKL algorithm to simultaneously learn the contribution of each brain region, previously defined by an atlas, to the decision function. As the considered technique is sparse, some kernels (here corresponding to brain regions) will have a perfectly null contribution to the final decision function. The resulting weight maps at the voxel and region levels will hence be sparse and do not need to be thresholded. In summary, here we investigate the introduction of anatomical or functional a priori knowledge in a MKL whole brain model and compare the results when using different atlases, both in terms of model performance and obtained weight maps. The proposed approach has two potential benefits: (1) it can lead to improved overall generalisation performance when the grouping structure imposed by the atlas is consistent with the data; (2) it can identify a subset of relevant brain regions for the predictive model. It is important to note that our approach does not provide information about why a specific feature has a high weight (or contribution) to the model (Haufe et al. 2014, Weichwald et al. 2015) but rather aims at identifying a (sparse) list of regions that contribute to the model’s predictive function. The approach is illustrated using three different atlases (described in the methods section) and three public datasets: the functional MRI (fMRI) Haxby dataset (Haxby et al. 2001) which investigates the differences in brain activity when viewing different types of visual stimuli, the fMRI ‘face’ data set (Henson et al. 2002) which studies changes in brain activity when looking at images of faces (famous, non-famous and scrambled), and the structural MRI (sMRI) OASIS dataset (Open-Access Series of Studies, oasis-brains.org; Marcus et al. 2007), which consists of structural images obtained from non-demented and demented older adults. The method was implemented in PRoNTo (http://www.mlnl.cs.ucl.ac.uk/pronto/).

## Materials and Methods

### Datasets and Pre-Processing

Three public datasets were used to illustrate the proposed approach. The first one has been previously used in pattern recognition for neuroimaging studies (Haxby et al. 2001; Hanson et al. 2004; O’Toole et al. 2005) and for describing the functionalities of different software toolboxes (Hanke et al. 2009a, b; Schrouff et al. 2013a). The data consist of a block design fMRI experiment acquired using a visual paradigm, where the participants passively viewed grey scale images of eight categories: pictures of faces, cats, houses, chairs, scissors, shoes, bottles, and control, non-sense images. As an illustrative example, we chose to analyse the data from a single subject (participant 1), consisting of 12 runs, each comprising eight blocks of 24 s showing one of the eight different object types and separated by periods of rest. Each image was shown for 500 ms followed by a 1500 ms inter-stimulus interval. Full-brain fMRI data were recorded with a volume repetition time of 2.5 s. Each category block therefore corresponds approximately to nine scans, separated by six scans of rest. For further information on the acquisition parameters, please consult the original reference (Haxby et al. 2001). The data were pre-processed using SPM8 (http://www. Fil.ion.ucl.ac.uk/spm/software/). We motion corrected, segmented and normalized the scans according to the MNI template. No smoothing was applied to the data.^3^ For proof of concept, we chose to focus the analysis on the comparison between viewing ‘faces’ and viewing ‘houses’, since it was reported as leading to high accuracy values and precise anatomical localization of the most discriminative regions (Schrouff et al. 2013a). Therefore we expected visual areas to have a high contribution to the predictive model.

The second dataset consisted of a single subject event-related fMRI data freely available from the SPM website comprising a repetition priming experiment, where two sets of 26 familiar (famous) and unfamiliar (non-famous) faces were presented against a checkerboard baseline. A random sequence of two presentations of each face was created from each set. The faces were presented for 500 ms with a stochastic distribution of stimulus onset asynchrony (SOA) determined by a minimal SOA of 4.5 s and 52 randomly interspersed null events. The subject was asked to make fame judgments by making key presses. Whole brain fMRI data were recorded with a volume repetition time of 2 s. For further information on the acquisition parameters, please consult the original work (Henson et al. 2002). The data were pre-processed using SPM8. This included motion correction, segmentation, normalization to the MNI template and smoothing ([8 8 8] mm). To classify famous versus non-famous faces we first fitted a GLM to all voxels within the brain, using SPM8. The design matrix comprised as many columns as events (all famous and non-famous faces presented, in order to obtain one beta image per event) plus the movement parameters and the mean regressor. The betas corresponding to the second repetition of famous and non-famous faces were used for classification.

The third dataset, the Open-Access Series of Studies (OASIS, oasis-brains.org; Marcus et al. 2007), illustrates the potential of the proposed methodologies in clinical settings. It consists of structural MRI images from non-demented and demented older adults. In the OASIS dataset, patients were diagnosed with dementia using the Clinical Dementia Rating (CDR) scale as either non-demented or with very mild to mild Alzheimer’s disease (Morris 1993). A global CDR of 0 indicates no dementia (healthy subjects) and a CDR of 0.5, 1, 2 and 3 represent very mild, mild, moderate and severe dementia respectively. The patients were age and gender matched with the controls, such that our analysis comprises the structural MRI images from fifty patients diagnosed with very mild and mild dementia (M = 75.3, SD = 6.8, 28 females) and fifty healthy controls (M = 75, SD = 6.7, 28 females). The OASIS data were also pre-processed using SPM8. The first step was to average all the repeats for each session followed by a grey matter segmentation, then, the segmented images were normalized and smoothed with a Gaussian kernel with a full width at half maximum (FWHM) of [8 8 8] mm.

Additional pre-processing was applied before the machine learning modelling. The data were linearly detrended (fMRI data only, polynomial detrend of order 1). In order to ensure that the MKL and SVM models were based on the same set of voxels we built one binary mask from each atlas. In the case of the fMRI data, the mask defined by the considered atlas, was applied to each image to select the voxels used as a feature in the modelling. In the case of the structural MRI data, we first selected voxels that had a probability of being located in grey matter equal or above 30% in all subjects and then applied a mask defined by the considered atlas to select the voxels. For all datasets a linear kernel was built for each region as defined by the considered atlas.

**Fig. 1:**
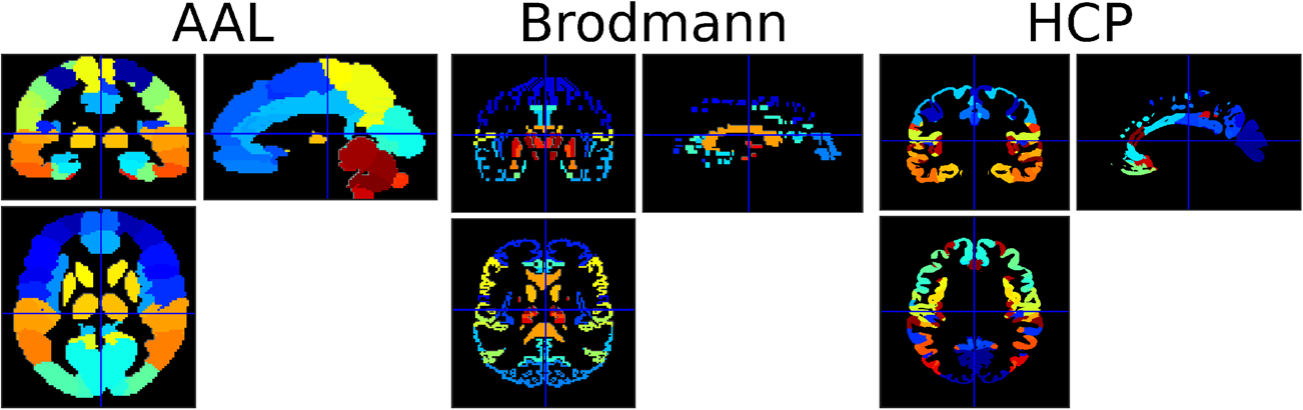
Illustration of the three atlases used, color-coded according to region numbers. The AAL atlas is displayed on the left, the Brodmann atlas in the middle and the HCP atlas on the right. Each atlas includes a different number of voxels and regions. Cross-hair positioned at [0,0,0]

Three atlases were used to investigate the effect of using different anatomical or functional priors in the MKL model (Fig. 1):

1. The Automated Anatomical Labeling **(**AAL, Tzourio-Mazoyer et al., 2002) atlas, built using the WFU-PickUp Atlas toolbox of SPM and consisting of 116 brain regions. This atlas is a widely used manual macroanatomical parcellation of the single subject MNI-space template brain.
2. The Brodmann + atlas, built using the WFU-PickUp Atlas toolbox of SPM and consisting of 75 regions. This atlas includes 47 out of the 52 areas defined by K. Brodmann, based on cytoarchitecture or histological structure, as well as other structures and nuclei.
3. The atlas built from the Human Connectome Project (HCP, Glasser et al. 2016). This multi-modal parcellation (atlas) is probably the most detailed cortical in-vivo parcellation available to date. The HCP MMP 1.0 has been built using surface-based registrations of multimodal MR acquisitions and an objective semi-automated neuroanatomical approach to delineate 180 areas per hemisphere bounded by sharp changes in cortical architecture, function, connectivity, and/or topography in a group average of 210 healthy young adults from the HCP cohort. It comprises 180 bilateral regions.

In all MKL models, the kernels were mean centred and normalized before classification, taking the training set/test set split into account. Mean centring the kernel corresponds to mean centre the features across samples (i.e. it is equivalent to subtracting the mean of each feature/voxel, computing the mean based on the training data), while normalizing the kernel corresponds to dividing each feature vector (i.e. each sample) by its norm. The later operation is particularly important when using MKL approaches to compensate for the fact that the different kernels might be computed from different numbers of features (i.e. different region sizes). This operation can hence be seen as giving an equal chance to all regions, independently of their sizes. Both operations were considered as pre-processing steps and can affect the model performance and obtained weight maps. For single kernel modelling (i.e. SVM models), the kernels were first added to provide the whole brain feature set, then mean centred. It should be noted that adding linear kernels is equivalent to concatenating the features/voxels. The resulting kernel is not normalized as this is not a common operation for single kernel modeling and often leads to decreases in model performance (unpublished results).

### Machine Learning, Modelling

The two classifiers considered in the present work are based on binary SVM machines (Boser et al., 1992). More specifically, single kernel analyses were conducted using the LIBSVM implementation of SVM (Chang and Lin 2011), while multi-kernel learning was performed using the SimpleMKL package (Rakotomamonjy et al., 2008), which resorts to the SimpleSVM algorithm (Canu et al., 2003). The framework of those two procedures is described below:

#### Single Kernel Modelling

Mathematically, let X∈R^n,l^, the data matrix of samples (*n*) by features (*l*) and y∈R^n^ the corresponding labels, where each row **x**_i_ corresponds to a feature vector and y_i_ corresponds to its respective label. Supervised learning approaches for binary classification, such as the SVM, estimate a decision function *f*, which separates the data into different classes defined by the labels. In a linear model, *f* is of the form (Eq. 2.1):

2.1 $$ f\left({\mathbf{x}}_{\mathrm{i}}\right)=\left\langle \mathbf{w},{\mathbf{x}}_i\right\rangle +b $$

With <,> representing the dot product between the weight vector **w** ∈ R^l^ and a feature vector **x**_i_, and *b* being a bias term.

The decision function *f* of an SVM is obtained by solving the following optimisation problem (Boser et al., 1992):

2.2 $$ {\displaystyle \begin{array}{c}\operatorname{minimize}\ \frac{1}{2}\parallel \mathbf{w}{\parallel}^2+C\sum \limits_i{\xi}_i\\ {}\mathrm{subject}\kern0.34em \mathrm{to}\kern0.70em {y}_i\left(\left\langle \mathbf{w},{\mathbf{x}}_i\right\rangle +b\right)\ge 1-{\xi}_i\kern0.75em \forall i\\ {}{\xi}_i\ge 0\kern0.75em \forall i\end{array}} $$

Where *i* indexes the samples, from 1 to *n*, *C* corresponds to the soft-margin parameter, ∑_*i*_*ξ*_*i*_ is an upper-bound on the number of training errors and *b* is a bias term. The solution of the optimisation problem can be written as (please see appendix A1 for details):

2.3 $$ \mathbf{w}={\sum}_{i=1}^n{y}_i{\alpha}_i{\mathbf{x}}_i $$

Substituting **w** into Eq. 2.1 and considering the linear kernel definition *K*(**x**, **x**_*i*_) = ⟨**x**, **x**_*i*_⟩, we can re-write the decision function in its dual form as

2.4 $$ f\left({\mathbf{x}}_i\right)={\sum}_{i=1}^n{\alpha}_iK\left(\mathbf{x},{\mathbf{x}}_i\right)+b $$

Where *α*_*i*_ and *b* represent the coefficients to be learned from the examples and *K*(**x**, **x**_*i*_), the kernel, is a function characterising the similarity between samples **x** and **x**_i_.

**Fig. 2:**
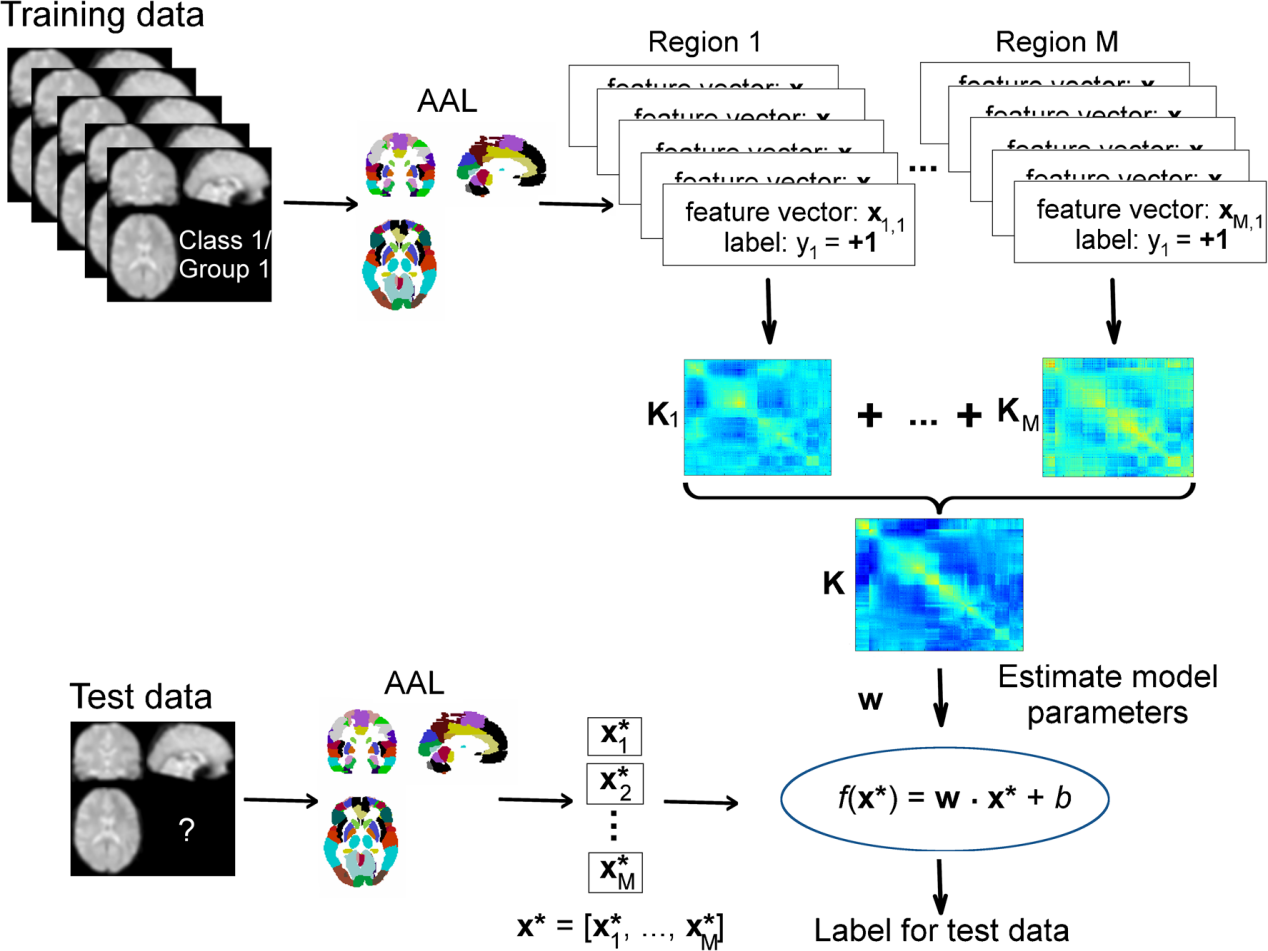
Illustration of the single kernel SVM classification procedure. For each image i, the signal in each voxel is extracted and concatenated in the feature vector **x**_**m,i**_ according to the M different regions defined by the AAL atlas. Each vector is associated to a label y_i_ (+1 or −1 in the case of binary classification). A linear kernel **K**_**m**_ is then built from the feature vectors for each region m (m = 1, …, M). The computed kernels **K**_**m**_ are added to obtain a whole brain linear kernel **K**. The kernel and its associated labels are used to train the model and estimate the model parameters **w**. The model can then be applied to new/unseen data x* to obtain an associated predicted label

An illustration of whole brain single kernel modelling is presented in Fig. 2.

#### Multiple Kernel Learning

In multiple kernel learning, the kernel *K*(**x**, **x**^′^) can be considered as a linear combination of *M* “basis” kernels (Lanckriet et al. 2004), i.e.:

2.5 $$ K\left(\mathbf{x},{\mathbf{x}}^{\prime}\right)={\sum}_{m=1}^M{d}_m{K}_m\left(\mathbf{x},{\mathbf{x}}^{\prime}\right),\kern.3em \mathrm{with}\kern.3em {d}_m\ge 0,{\sum}_{m=1}^M{d}_m=1 $$

Therefore, the decision function of an MKL problem can be expressed in the form:

2.6 $$ f\left({\mathbf{x}}_i\right)={\sum}_m\left\langle {\mathbf{w}}_m,{\mathbf{x}}_i\right\rangle +b $$

The considered multiple kernel learning approach is based on the primal formulation of an SVM binary classifier (Rakotomamonjy et al., 2008) and the solution can be obtained by solving the following optimisation problem:

2.7 $$ {\displaystyle \begin{array}{c}\operatorname{minimize}\kern0.5em \frac{1}{2}\sum \limits_m\frac{1}{d_m}\parallel {\mathbf{w}}_m\parallel {}^2+C\sum \limits_i{\xi}_i\\ {}\mathrm{subject}\kern0.34em \mathrm{to}\kern0.75em {y}_i\left(\sum \limits_m\left\langle {\mathbf{w}}_m,{\mathbf{x}}_i\right\rangle +b\right)\ge 1-{\xi}_i\kern0.5em \forall i\\ {}{\xi}_i\ge 0\kern0.5em \forall i\\ {}\sum \limits_m{d}_m=1,{d}_m\ge 0\kern0.5em \forall m\end{array}} $$

With *d*_*m*_ representing the contribution of each kernel *K*_*m*_ to the model. Therefore, both *d*_*m*_and **w**_*m*_ have to be learned simultaneously. In this formulation, proposed by (Rakotomamonjy et al., 2008), the L1 constraint on *d*_*m*_ enforces sparsity on the kernels with a contribution to the model. Furthermore, it results in a convex optimisation problem that can be solved using a simple SVM machine on *K* and gradient descents to find *d*_*m*_. For further details, please refer to (Rakotomamonjy, et al., 2008).

For the considered MKL optimisation problem the weights **w**_*m*_ can be expressed as (please see appendix A1 for details)

2.8 $$ {\mathbf{w}}_m={d}_m\sum \limits_{i=1}^n{y}_i{\alpha}_i{\mathbf{x}}_i $$

**Fig. 3:**
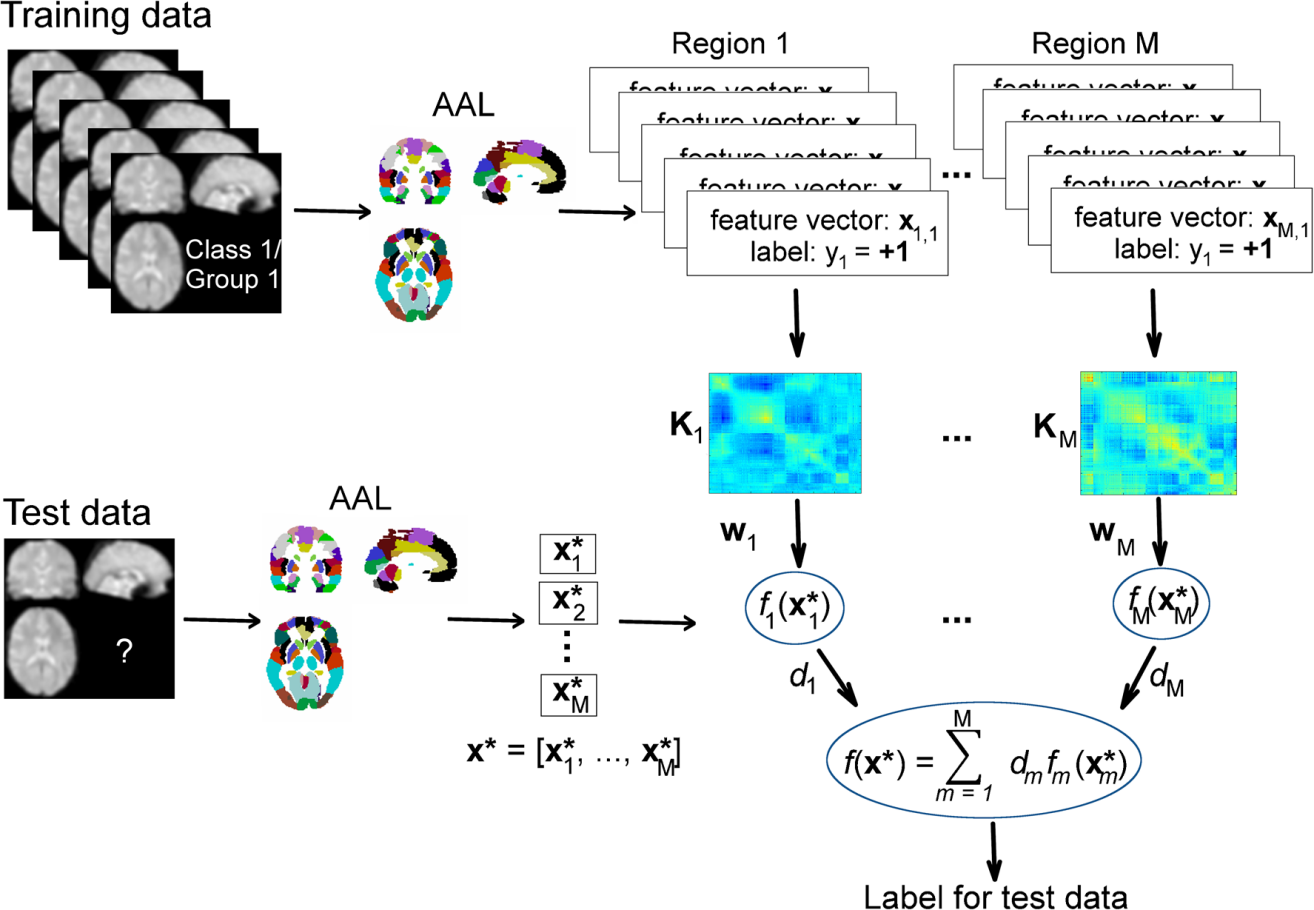
Illustration of the multiple kernel learning classification procedure. For each image i, the signal in each voxel is extracted and concatenated in the feature vector **x**_**m,i**_ according to the M different regions defined by the AAL atlas. Each vector is associated to a label y_i_ (+1 or −1 in the case of binary classification). A linear kernel **K**_**m**_ is then built from the feature vectors for each region m (m = 1, …, M). The kernels and their associated labels are used to train the model. First, model parameters **w**_**m**_ are estimated to define a decision function *f*_m_ per kernel. The weight of each decision function, d_m_, is then estimated to provide a final decision function *f*(**x**). The model can then be applied to new/unseen data x* to obtain an associated predicted label, based on feature vectors defined using the same atlas, **x**_1_*, **x**_2_*, …, **x**_M_*

In the present case, MKL can be seen as a feature selection technique, i.e. each kernel corresponds to a different subset of features (corresponding to the labelled regions). The considered approach is illustrated in Fig. 3. However, MKL can potentially be used as a model selection strategy, where each kernel corresponds to a different model (e.g. different parameter of a non-linear kernel, Rakotomamonjy et al., 2008). In a neuroimaging context, MKL approaches were mostly used to combine heterogeneous sources of features, such as different imaging modalities (e.g. Filippone et al., 2012) or imaging with psychological testing (e.g. Filipovych et al., 2011). Such combination of multiple image modalities can also be performed using the MKL implementation in PRoNTo v2.0.

#### Assessing Performance

We performed a nested cross-validation procedure to train the model and optimise the model’s hyperparameters. The external loop was used for assessing the model’s performance and the internal loop was used for optimising the models hyperparameters (soft-margin parameter, C, for the SVM and SimpleMKL). For all models (MKL and SVM) the hyperparameter range was [0.01, 1, 100]. The reason for the limited number of tested values was the high computational cost of MKL with parameter optimisation. For the Haxby dataset we used a *leave-one-block-out* cross-validation for the external loop and the internal loop. For the ‘face’ dataset, we performed a *leave-one-example-per-class-out* cross-validation, for the external and internal loop. For the OASIS dataset we used a *k-folds cross-validation on subjects-per-group*, with k = 10 folds for the external loop (i.e. leaving 10% of the subjects out, half of them being demented, half being healthy) and k = 5 folds for the internal loop. Model performance was assessed by balanced accuracy values, computed as the average of the class accuracies (corresponding to the sensitivity and specificity). A *p*-value was associated to each accuracy measure using permutation tests: the labels of the examples in the training set were randomly shuffled (taking the block structure of the datasets into account) before building a model. Results were considered significant when the obtained models performed equally or better than the model without shuffling the labels at most 5% of the time across 100 permutations (i.e. p-value < 0.05).

### Weight Map

As shown in Eq. 2.8 the models weights (**w** and **w**_*m*_), representing the contribution of each feature (here voxel) for the decision function or predictive model can be explicitly computed and plotted as brain images in order to display the decision function of the model based on previously defined brain regions. To avoid scaling issues between weight maps (e.g. from different folds or data sets), the resulting weight maps were normalized (i.e. **w**/||**w**||_2_).

As our MKL approach can be seen as a hierarchical model of the brain, it is possible to derive weights at two levels: (1) the weight or contribution of each region to the decision function, i.e. the values of *d*_*m*_, and (2) the weights for each voxel (see appendix A2 for the derivation of the weights per voxel). The weights at the voxel level can provide insights on the homogeneity of the discriminative patterns within the regions. Regions were ranked according to their contribution to the model (i.e. *d*_*m*_), averaged across folds. Only regions with a positive (i.e. non-null) contribution to the decision function *f* are displayed (i.e. #*d*_*m*_ > 0).

#### Stability of the Regions’ Contribution

To investigate whether the selected regions are stable across the folds of the cross-validation (i.e. variability in the training data), we computed the “reproducibility” of the regions’ ranking. Firstly, the ranking of a region is computed within each fold by sorting the kernel contributions in ascending order. Regions with a null contribution were assigned a null rank. The minimum value of the ranking is hence 0, while its maximum corresponds to the number of regions. The Expected Ranking (ER) is computed as the average of the ranking across folds. As in (Kia et al. 2016), we compute the cosine of the angle between the expected ranking (ER) and the ranking in each fold and estimate the ‘reproducibility’ of the ranking as the expectation of the cosine. This measure provides an estimation of the ‘distance’ between the ranking in each fold and the average ranking.

More specifically, if we assume an angle α_j_ between ER and R_j_, the ranking in fold j (j = 1… number of folds), we have (Eq. 2.9):

2.9 $$ \cos \left({\alpha}_j\right)=\kern0.5em \frac{ER\times {R}_j}{\parallel ER\parallel \times \parallel {R}_j\parallel } $$

The reproducibility ψ_R_ of the ranking (0 < =ψ_R_ < =1) is then (Eq. 2.10):

2.10 $$ {\psi}_R=E\left[\cos \left({\alpha}_j\right)\right],\kern0.5em \forall j=1\dots \mathrm{number}\  \mathrm{of}\  \mathrm{folds} $$

The closer this number is to 1, the more stable the solution is across folds. It is important to note that these values are meaningful only if the corresponding model performs significantly above chance level.

#### Comparison of Atlases

We finally compare different priors (i.e. atlases) in terms of obtained weight maps. To this end, we computed the Pearson correlation between the weight maps at the voxel level of each atlas, for overlapping voxels (i.e. voxels considered for modeling in both atlases). We then obtained three values of correlation, one for each pair of atlas. The closer this value is to one, the more similar the two considered weight maps are. The significance of the obtained correlation values was tested using 1000 non-parametric permutations. As correlation measures do not take into account null values, we also estimated the proportion of null weights that is shared by both atlases (i.e. voxels with 0 weight in both atlases, the intersection of null values) compared to the total number of overlapping voxels.

## Results

### Haxby Dataset

#### Model Performance

Table 1 shows that the model can discriminate with high accuracy if the subject was viewing images of faces versus images of buildings, for all models and atlases. This was expected in view of the previous performances obtained using this dataset (e.g. Hanke et al. 2009a; Schrouff et al. 2013a). Overall, the MKL models perform better than the SVM models, with the MKL-HCP model leading to the best performance. For both MKL and SVM, the Brodmann atlas leads to a slight decrease in balanced accuracy when compared to the AAL and HCP atlases. Please note that using only the left and right fusiform regions as defined by the AAL atlas leads to a balanced accuracy of 99.5% (108/108, 107/108). This shows that these visual areas carry a lot of predictive information. These regions are therefore expected to have a high model contribution and ER.

**Table 1:** Model performance for the MKL and SVM models distinguishing between ‘faces’ (F) and ‘houses’ (H), for each atlas (in %, with *p*-value)

Model	Atlas	Balanced accuracy (%)	True positives (Faces)/ Total positives	True negatives (Houses)/ Total negatives
MKL	AAL	98.15 (*p* = 0.01)	107/108	105/108
	Brodmann	96.30 (*p* = 0.01)	104/108	104/108
	HCP	100.0 (*p* = 0.01)	108/108	108/108
SVM	AAL	93.06 (*p* = 0.01)	101/108	100/108
	Brodmann	91.20 (*p* = 0.01)	96/108	101/108
	HCP	94.91 (*p* = 0.01)	100/108	105/108

True positives (resp. negatives) represent the class accuracy for faces (resp. houses) samples classified correctly as faces (resp. houses). Note that the difference between the SVM models is only the mask used to select the voxels, which is based on the atlas

#### Stability of the Regions’ Contribution

For each MKL model, we present the number of regions selected (i.e. with a non-null contribution across folds) in Table 2, as well as the model’s reproducibility.

**Table 2:** Number of regions selected across folds and model reproducibility, for each MKL model

Model	Atlas	ROIs (/total)	Reproducibility
MKL	AAL	14 (/116)	0.9415
	Brodmann	21 (/74)	0.8690
	HCP	13 (/180)	0.9396

For this dataset, all models are quite sparse, with a relatively low number of regions with a non-null contribution to the model. The models with the highest accuracies (namely AAL and HCP) also lead to the highest reproducibility.

#### Comparison of Weight Maps Across Atlases

**Fig. 4:**
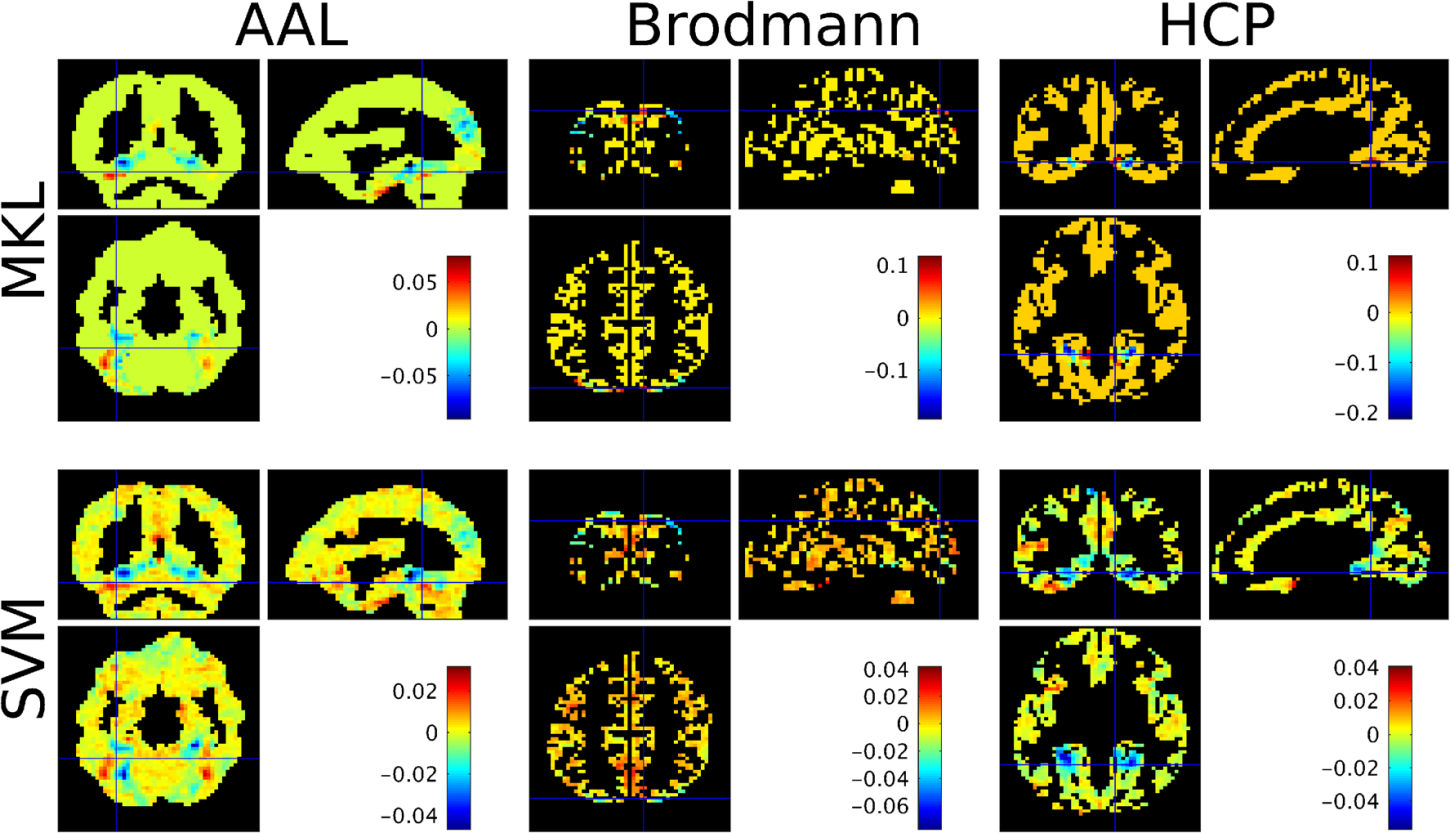
Weight images at the voxel level for the ‘faces’ versus ‘houses’ comparison based on the Haxby dataset (average across folds), for each model considered. For the MKL models (top row), voxels in green (AAL), yellow (Brodmann) or orange (HCP) have a null contribution to the model. For each atlas, the cross-hair was positioned on the region with the highest MKL model contribution across folds (i.e. *d*_*m*_)

The weight maps for each atlas (at the voxel level) are displayed in Fig. 4 and the list of selected regions with non-null contributions for the MKL models for each atlas are displayed in appendices Tables 7, 8 and 9, along with their contributions *d*_*m*_ and expected ranking ER. We can see that the fusiform regions (left and right) are ranked highly in the MKL-AAL model (ranks 115/116 and 103/116, respectively). Similarly, the MKL-Brodmann model selected area 19 (visual cortex, V3, V4 and V5) with highest rank (70/74), and area 37 (overlapping with the fusiform gyrus) with rank (50/74). In contrast, the MKL-HCP model selected ventromedial areas 1 and 2 with ranks (180/180) and (170/180), respectively.

In order to verify if the weight maps for the different MKL models were similar we computed the pairwise correlation coefficient between the weight vectors for the different models. The weight vectors for the AAL-MKL and Brodmann-MKL models have a correlation coefficient of ρ = 0.5330 (*p* = 9.9e^−4^), with 79.16% of voxels with a null weight in both models. The AAL-MKL and HCP-MKL weight vectors have a correlation coefficient of ρ = 0.3470 (*p* = 9.9e^−4^) and shared 83.05% of null weights, while the Brodmann-MKL and HCP-MKL weight vectors have a correlation coefficient of ρ = 0.5402 (*p* = 9.9e^−4^) with 86.51% of common null weights. The weight vectors for the two models leading to the highest performance and reproducibility are hence significantly correlated.

### Face Dataset

#### Model Performance

Table 3 displays model performance for the MKL and SVM models considered. Most of the models were able to discriminate if the subjects were looking at ‘famous’ vs ‘non-famous faces’, however the accuracies were lower than the ones observed for the Haxby dataset. For this dataset there is an improvement in performance for the MKL models based on the AAL and Brodmann atlases with respect to the SVM models. The Brodmann-MKL model has the best performance across the MKL models. Results for the HCP-MKL and AAL-SVM models are not significant.

**Table 3:** Model performance for the MKL and SVM whole brain models distinguishing between ‘famous faces’ (F) and ‘non-famous faces’ (N)

**Model**	Atlas	Balanced accuracy (%)	True positives (F)/ Total positives	True negatives (N)/ Total negatives
**MKL**	AAL	73.08 (*p* = 0.01)	20/26	18/26
	Brodmann	75.00 (*p* = 0.02)	19/26	20/26
	HCP	67.31 (*p* = 0.11)	19/26	16/26
**SVM**	AAL	65.38 (*p* = 0.05)	17/26	17/26
	Brodmann	67.31 (*p* = 0.04)	18/26	19/26
	HCP	67.31 (*p* = 0.04)	18/26	18/26

True positives (resp. negatives) represent the class accuracy for ‘famous faces’ (resp. ‘non-famous faces’) samples classified correctly as ‘famous faces’ (resp. ‘non-famous faces’). Note that the difference between the SVM models is only the mask used to select the voxels, which is based on the atlas

#### Stability of the Regions’ Contribution

For each MKL model, we present the number of regions selected (i.e. with a non-null contribution across folds) in Table 4, as well as the model’s reproducibility.

**Table 4:** Number of regions selected across folds (compared to the total number of regions considered) and model reproducibility, for each MKL model

Model	Atlas	ROIs (/total)	Reproducibility
MKL	AAL	37 (/105)	0.8846
	Brodmann	33 (/69)	0.8830
	HCP	66 (/180)	0.8091

For this dataset, between 35% and 48% of the regions were selected, resulting in moderate sparsity. As for the Haxby dataset, the atlases leading to the best performance (namely AAL and Brodmann) lead to the highest reproducibility.

#### Comparison of Weight Maps Across Atlases

**Fig. 5:**
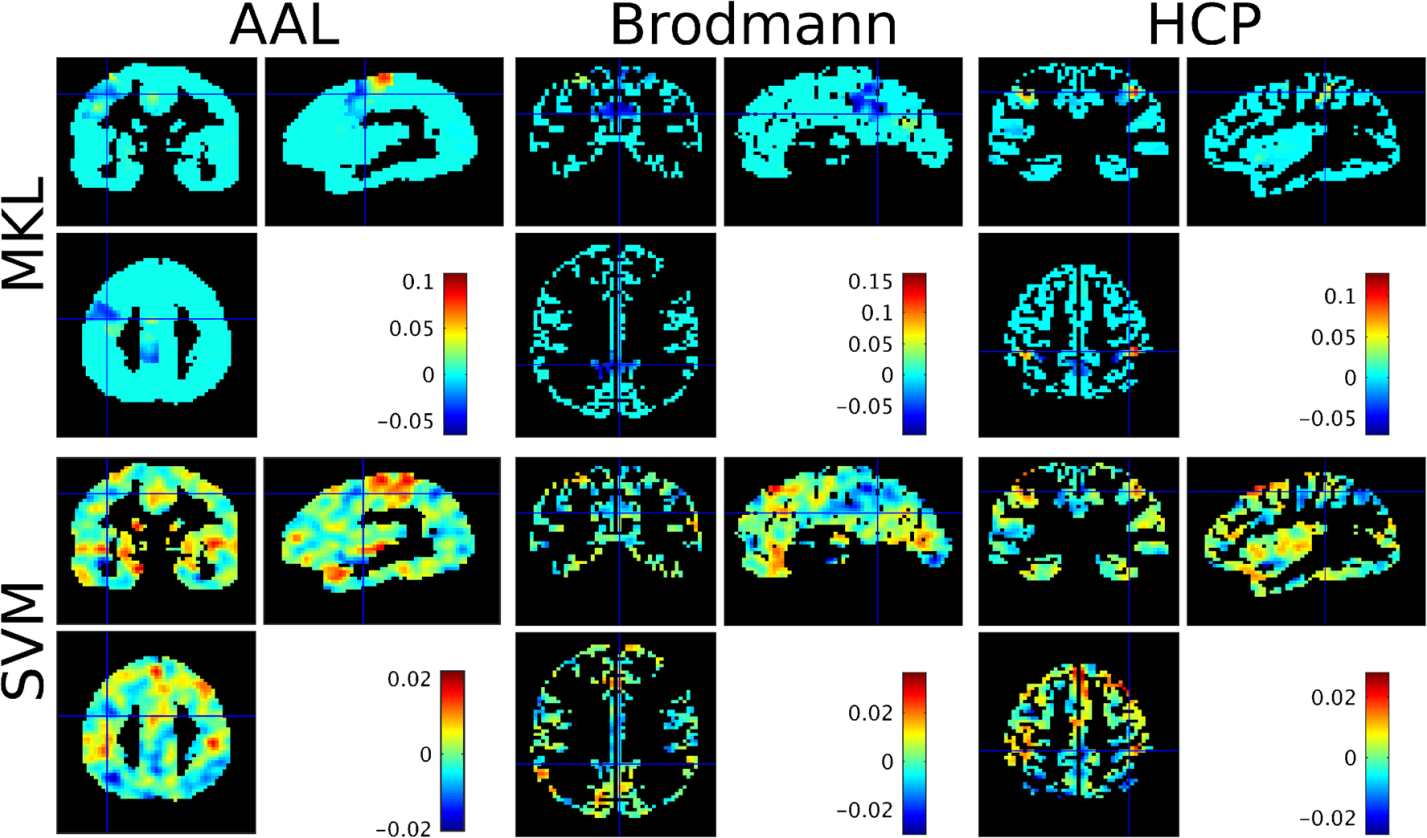
Weight images at the voxel level for the ‘famous’ versus ‘non-famous’ comparison based on the “faces” dataset (average across folds), for each model considered. For the MKL models (top row), voxels in light blue have a null contribution to the model. For each atlas, the cross-hair was positioned on the region with the highest MKL model contribution across folds (i.e. *d*_*m*_)

The weight maps for each atlas (at the voxel level) are displayed in Fig. 5 and the list of selected regions with non-null contributions for the MKL models for each atlas are displayed in appendices Tables 10, 11 and 12, along with their contributions *d*_*m*_ and expected ranking ER. The regions with the highest expected rankings in the AAL-MKL model were the precentral gyrus (ER = 105/105), the cingulum (ER = 104/105), the occipital gyrus (ER = 99/105), the pallidum (ER = 99/105) and the inferior frontal cortex (ER = 98/105). The MKL-Brodmann model selected areas 31 (portion of the posterior cingulate cortex, ER = 68/69), 5 (primary somatosensory cortex, ER = 67/69), substantia nigra (ER = 66/69), 1 (postcentral gyrus, ER = 65/69) and 44 (inferior frontal gyrus, ER = 65/69) with highest expected rankings. The MKL-HCP model selected somatosensory cortex (Area 2, ER = 180/180), precuneus (ER = 177/180) and premotor regions (Dorsal area 6, ER = 176/180) with highest expected ranking.

The correlation coefficient between the weight vectors for the AAL-MKL and Brodmann-MKL models is ρ = 0.1717 (*p* = 9.9e^−4^), with 34.23% of voxels with a null weight in both atlases. The AAL-MKL and HCP-MKL weight vectors have a correlation coefficient of ρ = 0.2760 (*p* = 9.9e^−4^) and shared 44.43% of null weights, while the Brodmann-MKL and HCP-MKL weight vectors have a correlation coefficient of ρ = 0.1550 (*p* = 9.9e^−4^) with 33.00% of common null weights. For this dataset, the similarity between weight maps is much lower than for the Haxby dataset, with most null weights being so in only one atlas.

### OASIS

#### Model Performance

Classifying healthy versus demented patients (with mild and very mild dementia) led to the accuracy values presented in Table 5. All models led to significant classification results. The results show that SVM models perform better than the MKL models for this dataset, and that the Brodmann atlas led to highest performance for both MKL and SVM models.

**Table 5:** Model performance for the MKL and SVM whole brain models distinguishing between ‘demented patients’ (D) and ‘control’ (C)

Model	Atlas	Balanced accuracy (%)	True positives (D)/ Total positives	True negatives (C)/ Total negatives
MKL	AAL	66.00 (*p* = 0.01)	34/50	32/50
	Brodmann	68.00 (*p* = 0.01)	34/50	34/50
	HCP	65.00 (*p* = 0.01)	34/50	31/50
SVM	AAL	67.00 (*p* = 0.01)	32/50	35/50
	Brodmann	70.00 (*p* = 0.01)	33/50	37/50
	HCP	63.00 (*p* = 0.01)	29/50	34/50

True positives (resp. negatives) represent the number of demented (resp. non-demented) patients classified correctly as demented (resp. non-demented). Note that the difference between the SVM models is only the mask used to select the voxels, which is based on the atlas

#### Stability of the Regions’ Contribution

For each MKL model, we present the number of regions selected (i.e. with a non-null contribution across folds) in Table 6, as well as the model’s reproducibility.

**Table 6:** Number of regions selected across folds and model reproducibility, for each MKL model

Model	Atlas	ROIs (/total)	Reproducibility
MKL	AAL	73 (/116)	0.7769
	Brodmann	46 (/65)	0.8862
	HCP	85 (/180)	0.7767

The decision function seems to be based on a more distributed set of regions for this dataset. This was also supported by the higher model performance of SVM compared to MKL, since the SVM is a non-sparse model. As observed in the other datasets, the model leading to the highest accuracy (i.e. using Brodmann atlas) leads to the highest reproducibility.

#### Comparison of Weight Maps Across Atlases

**Fig. 6:**
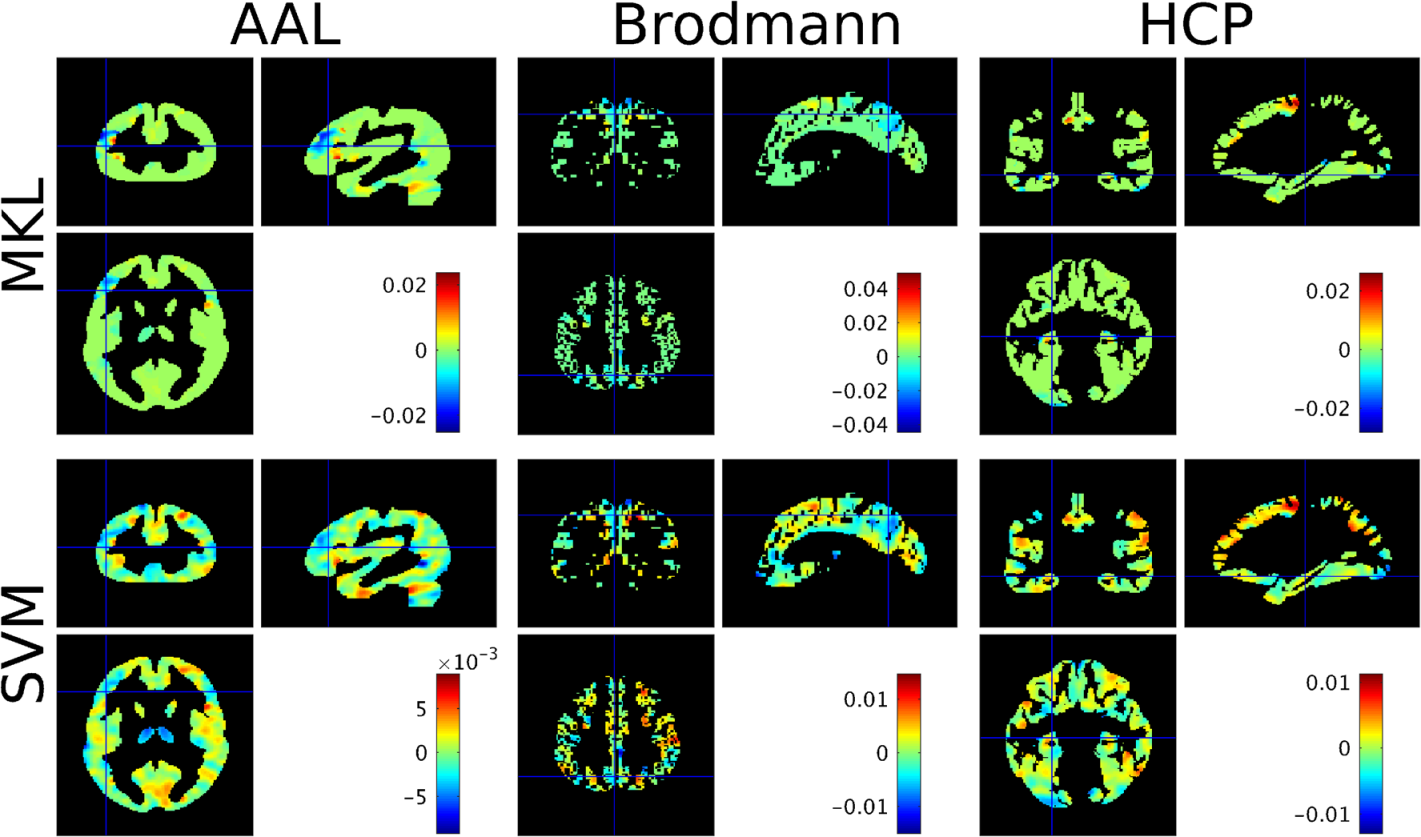
Weight images at the voxel level for the ‘demented’ versus ‘non-demented’ comparison based on the Oasis dataset (average across folds), for each model considered. For the MKL models (top row), voxels in green have a null contribution to the model. For each atlas, the cross-hair was positioned on the region with the highest MKL model contribution across folds (i.e. *d*_*m*_)

The weight maps for each atlas (at the voxel level) are displayed in Fig. 6 and the list of selected regions with non-null contributions for the MKL models for each atlas are displayed in appendices Tables 13, 14 and 15, along with their contributions *d*_*m*_ and expected ranking ER. The regions with highest ranks in the MKL-AAL model include frontal regions (ER = 115/116), lingual gyrus (ER = 112/116), thalamus (ER = 108/116) and precuneus (ER = 101/116). The hippocampi were ranked 89/116 for right hippocampus and 61/116 for left hippocampus. The MKL-Brodmann model selected areas 7 (including the precuneus, ER = 63/69), 46 (including parts of the middle and inferior frontal gyrus, ER = 62/69), and 6 (premotor cortex and supplementary motor area, ER = 62/69) with high ER. The hippocampus had an expected ranking of 21/65. The regions with highest expected ranking according to the MKL-HCP regions were the hippocampus (ER = 178/180), posterior cingulate cortex (Area 23c, ER = 176/180) and part of lateral temporal cortex (Area TE2 anterior, ER = 152/180).

The correlation coefficient between the weight vectors at the voxel level for the AAL-MKL and Brodmann-MKL models is ρ = 0.4213 (*p* = 9.9e^−4^), with 7.79% of voxels with null weights in both atlases. The AAL-MKL and HCP-MKL weight vectors have a correlation coefficient of ρ = 0.3507 (*p* = 9.9e^−4^) and shared 18.15% of null weights, while the Brodmann-MKL and HCP- weight vectors have a correlation coefficient of ρ = 0.4632 (*p* = 9.9e^−4^) with 9.43% of common null weights. For this dataset, there were more similarities between the AAL-MKL and the Brodmann-MKL models and between the Brodmann-MKL and the HCP-MKL models than between the AAL-MKL and the HCP-MKL models, which both have lower accuracies.

## Discussion

In this work, we present a novel approach to introduce anatomical or functional information in whole-brain machine learning models. Our procedure combines a priori information about the brain anatomy or function from an atlas with Multiple Kernel Learning (MKL, Rakotomamonjy et al., 2008), thereby estimating the contribution of each previously defined region of interest for the predictive model. Furthermore, the considered algorithm is sparse in the number of kernels (L1-norm constraint), therefore it selects a subset of regions that carry predictive information. Our approach results in a list of pre-defined brain regions, which can be ranked according to their contribution to the model. As previously mentioned, the obtained list of regions does not need to be thresholded since the regions which were not selected by the model in any fold have a null contribution to the model (i.e. *d*_*m*_ = 0). This is a clear asset over techniques such as summarising region weights post-hoc (Schrouff et al., 2013b) or the locally multivariate searchlight approach (Kriegeskorte et al., 2006). In the proposed approach, there is indeed no need to apply statistical tests to select regions with significant contributions and to apply corrections for multiple comparisons.

Our results show that the MKL model combining anatomically or functionally labelled regions had higher performance in comparison with the SVM model for the two fMRI datasets considered but not for the structural one (OASIS). These results suggest that the model assumptions of the MKL implementation considered (i.e. only a small subset of regions carry predictive information) is adequate for the fMRI datasets but not for the OASIS dataset. However, it is important to notice that the MKL results are also affected by the choice of the atlas (or the grouping structure), with some atlas being better than others depending on the dataset considered. The HCP atlas led to the best performance for the Haxby dataset, the AAL led to the best performance for the faces data set, while the Brodmann atlas led to best performance for the structural dataset. There was also a difference in performance for the SVM model as the voxels included for modeling are different from one atlas to another.

In terms of selected regions, the list of selected regions was dependent on the choice of the atlas. The different atlases considered have different brain coverage and very different parcellation of regions, therefore differences in the selection of regions are expected. In addition, the sparsity constraint of the MKL model might also contribute for the difference between the selected regions across atlases, as regions with correlated information might not be selected as being relevant for the predictive model.

As previously mentioned in the introduction, and discussed in previous works (Haufe et al., 2014, Weichwald et al., 2015), multivariate predictive models cannot provide a clear answer to why a specific region/feature has a high contribution to the model. Alternative approaches (e.g. univariate tests, correlation analyses, …) should be used to investigate why a set of regions has predictive information. In this work, we assess whether the highest-ranking regions are ‘meaningful’ by referring to the literature on the cognitive neuroscience and clinical issues they tackle.

Considering the Haxby dataset, the MKL models were able to discriminate with high accuracy if the subject was viewing images of faces versus images of buildings regardless of the atlas used. All MKL models identified regions that comprise the core system for visual processing as informative (Haxby et al., 2000 and Haxby et al. 2001). Nonetheless, the HCP atlas led to the best performance, reaching 100% balanced accuracy. The visual brain region with the highest contribution for HCP-MKL model was the ventro-medial visual area on the ventral surface of each hemisphere. It should be noted that this region has not been previously well parcellated in either the human or the macaque, usually being either left unparcellated or parts of it being included in other areas (Glasser et al., 2016). Surprisingly, the HCP-MKL was not able to select the fusiform face area. The way the visual system is differently parcellated in the three atlases might explain the differences observed in the balanced accuracy for the MKL models based on different atlases. For example, while the fusiform gyrus, selected as a region with high contribution by AAL-MKL model, is a relatively large region, in contrast, the HCP atlas provides a more detailed segmentation of the visual areas into smaller regions.

For the face dataset, both the AAL-MKL and the Brodmann-MKL models were able to discriminate if the subjects were looking at famous faces versus unfamiliar faces. Surprisingly, the results for the HCP-MKL were not statistically significant according to the permutation test. A possible explanation for these results is the very small sample size (only 26 images per class), which can lead to very high variance in the model’s performance, particularly when the leave-one-out cross validation framework is used (Varoquaux et al., 2017). Overall, the AAL-MKL model was able to identify regions that play an important role in visual processing such as cuneus, occipital regions and lingual gyrus and the Brodmann-MKL model was able to identify visuospatial area (BA7) and somatosensory association cortex (Minnesbusch et al., 2009; Liu et al. 2014). The selected regions for both atlases also included regions that have been implicated in recollection of episodic memories such as the precuneus and the posterior cingulate, (Nielson et al., 2010). In addition, prefrontal regions including the dorsolateral and ventromedial prefrontal cortex for Brodmann and the orbitofrontal cortex for both atlases were also selected. These regions have been found to be important for processing famous faces (Nielson et al., 2010; Sergerie et al. 2005; Leveroni et al., 2000), and may relate to the search and retrieval of person identity semantic information. The Brodmann-MKL model led to better performance than the AAL-MKL model. Interestingly, the posterior cingulate cortex presented the highest weight in the Brodmann-MKL model with a contribution of 21.92% for the predictive model. The posterior cingulate has been implicated in recollection of episodic memories (Henson et al. 1999; Maddock et al. 2001), and consistently reported as involved in the processing of famous faces (Leveroni et al., 2000; Nielson et al., 2010) therefore it might have an important role in accessing information about famous people. In contrast, the Brodmann-MKL model attributed high weights to motor regions (precentral and mid-cingulate) suggesting that different types of faces might prompt different patterns of motor responses, which might be related to differences in reaction time between the two tasks (subjects were asked to make fame judgements during the two conditions, ‘famous’ and ‘non-famous faces’). It should be noted that there were also similarities between the regions selected by the AAL-MKL and Brodmann-MKL, for example, the posterior cingulate cortex was selected by the AAL-MKL and the motor regions were selected by Brodmann-MKL. In summary, the face dataset presents a high variability between the top regions selected by the different MKL models depending on the atlas used. One possible explanation for this variability is that the classification task considered is more complex than the one presented in the Haxby dataset and therefore might involve a large network of regions, which are differently parcellated in the different atlases.

For the OASIS dataset, our results show that the MKL models were able to discriminate with moderate accuracy between anatomical brain scans of patients with mild and very mild dementia and brain scans of age/gender-matched healthy controls. These results are in agreement with previous Alzheimer’s literature, which shows that pattern recognition methods applied to structural MRI can consistently discriminate between brain scans of patient and healthy controls (Arimura et al., 2008, Klöppel et al., 2008, Magnin et al., 2009, Duchesne et al., 2008, Vemuri et al., 2008, Gerardin et al., 2009, Nho et al., 2010, Oliveira et al., 2010, Farhan et al., 2014). As expected, the MKL model was able to identify, for all atlases, regions that comprise the core system for episodic memories (including temporal regions, hippocampus, posterior cingulate and precuneus) and parieto-frontal regions, which are also in agreement with existing literature (Zhang et al., 2015, Magnin et al., 2009). The Brodmann atlas led to the highest performance, suggesting that this atlas has a good coverage and segmentation of the most discriminative regions. In the Brodmann-MKL model, the most informative region was the BA 7, which comprises the superior parietal lobule and part of precuneus. These regions have been described as diagnostic markers of Alzheimer’s disease (Karas et al., 2007, Quiroz et al. 2013). Surprisingly, the Brodmann-MKL did not select the hippocampus as one of the most relevant regions for the prediction. In contrast, the hippocampus has been selected as the region with the highest contribution to the predictive model by the HCP-MKL and the left hippocampus has been selected as the third region with the highest contribution by the AAL-MKL. One possible explanation for the Brodmann-MKL model not selecting the hippocampus as a highly informative region might be due to the limitation of the considered MKL model, which might not select two regions as being important if they have correlated information.

The considered MKL approach comprises a sparsity constraint on the kernels. This reflects an assumption that only a few kernels (or regions) carry predictive information, which might not be suited for all datasets. Our results show that the solutions for the three datasets considered had different degrees of sparsity. This might reflect that the pattern of interest is sparse for the Haxby and faces datasets, but not for the OASIS dataset. To dampen this issue, other regularization constrains, less conservative than the L1 could be envisaged. Future MKL developments including a combination of L1 and L2 regularizations should address this limitation. Our results also show that there was not an optimal atlas for all considered datasets. The definition of the atlas could also be approached as an optimisation problem, with the best grouping of regions being learnt automatically from the data.

In addition to identifying a subset of regions that contribute to the predictive model we can also investigate whether the selected results are stable across folds, i.e. is the selected subset of regions similar for slightly varying training sets? To investigate the stability of the selection of regions across folds, we used the expected ranking (ER) to estimate the reproducibility as a metric of stability. Across datasets, it seems that there is an association between reproducibility and how easy the discrimination between the categories is. This is illustrated by higher reproducibility score, ψ_R,_ for the Haxby dataset (average across atlases: 0.9181), lower for the ‘face’ dataset (average across atlases: 0.8523) and the lowest for the OASIS dataset (average across atlases: 0.8174). These results suggest that, when the sparse constraint is appropriate for the classification problem, the L1 MKL model leads to both high performance and high reproducibility (e.g. Haxby dataset). For the face dataset, there seems to be balance between performance and reproducibility. This last result is in agreement with Kia et al. 2016, who showed a similar effect during model optimisation across most subjects of the MEG recordings of the face data set. For the OASIS dataset, the model leading to the highest performance also led to the highest reproducibility. However, as mentioned above, the sparse prior does not seem appropriate for this dataset, which prevents us from drawing further conclusions. A potential improvement of our approach would be to include the reproducibility of the expected ranking of regions as a criterion for optimising the soft-margin hyperparameter (C), in addition to the generalization performance. The advantages of introducing reproducibility as an additional optimisation criterion has been also discussed in (Rosa et al. 2015; Kia et al. 2016 and Baldassarre et al. 2017).

### Implementation

The proposed MKL framework modelling the whole brain multivariate pattern as a combination of regional patterns has been implemented in our open-source software PRoNTo v2.0. The MKL algorithm corresponds to the simpleMKL toolbox (Rakotomamonjy et al., 2008). Detailed experiments on the memory usage and computational expenses can be found in the reference (Rakotomamonjy et al., 2008). Regarding memory use, the software (here Matlab) needs to be able to load all the ROI kernels simultaneously. Therefore, the size of the kernel (i.e. number of examples x number of examples) will play a role, as will the number of kernels built. Regarding CPU time, (Rakotomamonjy et al., 2008) showed that both the number of examples and the number of kernels affected computational expenses. For all the models considered in this work, running permutations to assess model significance was computationally expensive and was performed on a cluster for efficiency.

PRoNTo v2.0 also includes the post-hoc summarization of weights, as detailed in (Schrouff et al., 2013b). Nevertheless this approach suffers from the various limitations in terms of interpretation that were discussed in this work (i.e. the obtained list of regions should not be thresholded and the weights reflect the decision function of the model, not the neural sources of the signal).

Finally, PRoNTo v2.0 is provided under the GNU/GPL license ‘as is’ with no warranty. Our team has done its best to provide a robust framework to perform machine learning modeling of neuroimaging data. Such an endeavor is however a continuous process and we thank our users for reporting bugs. Improvements and bug fixes will be implemented in future versions of the software (v2.1 and v3.0, in progress).

In conclusion, here we present a new tool for introducing anatomical or functional information in whole-brain machine learning models using sparse multiple kernel learning. When the grouping structure defined by the atlas is consistent with the data and the sparsity constraint is appropriate, the proposed approach can lead to an increase in model performance when compared to whole-brain models. Furthermore, the obtained list of regions contributing to the model can then be investigated in terms of cognitive or clinical neuroscience and reproducibility.

### Information Sharing Statement

PRoNTo (RRID:SCR_006908) and the preprocessed data displayed in this work can be found at http://www.mlnl.cs.ucl.ac.uk/pronto/. The raw data can be accessed at: Haxby data set (https://openfmri.org/dataset/ds000105/), Faces data set (http://www.fil.ion.ucl.ac.uk/spm/data/), OASIS data set (http://www.oasis-brains.org/). The data was preprocessed using SPM (RRID:SCR_007037), which can be found at http://www.fil.ion.ucl.ac.uk/spm/software/. For modelling, we used the LIBSVM library (RRID:SCR_010243) to perform SVM classification (http://www.csie.ntu.edu.tw/~cjlin/libsvm/), while multi-kernel learning was performed using the SimpleMKL package (Rakotomamonjy et al., 2008, http://asi.insa-rouen.fr/enseignants/~arakoto/code/mklindex.html). Other software performing pattern recognition of neuroimaging data can be found at: The Decoding Toolbox (Hebart et al., 2015, http://www.bccn-berlin.de/tdt), MVPA toolbox (http://code.google.com/p/princeton-mvpa-toolbox/), PyMVPA (Hanke et al., 2009a, b, http://www.pymvpa.org/), Nilearn (Abraham et al., 2014, http://nilearn.github.io/), Representational Similarity Analysis (Kriegeskorte et al., 2008, http://www.mrc-cbu.cam.ac.uk/methods-and-resources/toolboxes/), CoSMoMVPA (Oosterhof et al., 2016, http://cosmomvpa.org/), Searchmight (Pereira and Botvinick, 2011, http://www.princeton.edu/~fpereira/searchmight/), 3Dsvm (Laconte et al., 2005, http://afni.nimh.nih.gov/pub/dist/doc/program_help/3dsvm.html), Probid (http://www.kcl.ac.uk/iop/depts/neuroimaging/research/imaginganalysis/Software/PROBID.aspx), Mania (Grotegerd et al., 2014, https://bitbucket.org/grotegerd/mania), PETRA (http://sipba.ugr.es/descargas/).

## Appendix A: Weights Derivations

### Support Vector Machine (SVM)

The Lagrangian of the SVM problem (Eq. 2.2.) is given by

$$ L=\frac{1}{2}\parallel \mathrm{w}\parallel {}^2+C{\sum}_i{\xi}_i+{\sum}_i{\alpha}_i\left(1-{\xi}_i-{y}_i\left(\left\langle \mathbf{w},{\mathbf{x}}_i\right\rangle +b\right)\right)-{\sum}_i{\nu}_i{\xi}_i $$

Setting to zero the derivatives of the Lagrangian with respect to **w**_m_ we get

$$ \frac{\partial L}{\partial \mathrm{w}}=\mathbf{w}-{\sum}_i{\alpha}_i{y}_i{\mathbf{x}}_i=0 $$

$$ \mathbf{w}={\sum}_i{\alpha}_i{y}_i{\mathbf{x}}_i $$

### Multiple Kernel Learning (MKL)

The Lagrangian of the MKL problem (Eq. 2.7.) is given by

$$ L=\frac{1}{2}{\sum}_m\frac{1}{d_m}\parallel {\mathbf{w}}_m\parallel {}^2+C{\sum}_i{\xi}_i+{\sum}_i{\alpha}_i\left(1-{\xi}_i-{y}_i\left({\sum}_m\left\langle {\mathbf{w}}_m,{\mathbf{x}}_i\right\rangle +b\right)\right)-{\sum}_i{\nu}_i{\xi}_i+\lambda \left({\sum}_m{d}_m-1\right)-{\sum}_m{n}_m{d}_m $$

Setting to zero the derivatives of the Lagrangian with respect to **w**_m_ we get

$$ \frac{\partial L}{\partial {\mathbf{w}}_m}=\frac{1}{d_m}{\mathbf{w}}_m-{\sum}_i{\alpha}_i{y}_i{\mathbf{x}}_i=0 $$

$$ {\mathbf{w}}_m={d}_m{\sum}_i{\alpha}_i{y}_i{\mathbf{x}}_i $$

## Haxby Data

### Regions Selected in MKL-AAL

Table 7 displays the regions with non-null contributions to the MKL-AAL model, along with their contribution *d*_*m*_, size (in voxels) and expected ranking ER, across folds.

**Table 7:** MKL modelling of the comparison of ‘faces’ vs ‘houses’, based on the AAL atlas

Region	Contribution (%)	Size	ER
Fusiform_L	21.342208	617	115.29
Occipital_Mid_L	20.902485	839	114.92
Lingual_R	20.323188	642	114.50
Lingual_L	13.591652	597	108.29
Fusiform_R	10.874692	687	102.96
Cingulum_Post_L	5.947777	143	110.83
Frontal_Inf_Oper_R	4.831543	373	101.17
Parietal_Sup_L	1.248777	401	81.54
Occipital_Sup_L	0.464520	314	45.17
Caudate_L	0.152138	285	4.58
Occipital_Mid_R	0.136176	441	9.00
Hippocampus_R	0.109758	284	13.54
Cingulum_Ant_L	0.074190	402	18.00
Thalamus_L	0.000894	290	4.50

Regions ranked further (i.e. rank >14) have a perfectly null contribution to the model. ER stands for Expected Ranking. The region size is displayed in voxels. The weights were averaged across folds (except for computing ER). A region label ending in ‘L’ (resp. ‘R’) means left (resp. right) hemisphere region

### Regions Selected in MKL-Brodmann

Table 8 displays the regions with non-null contributions to the MKL-Brodmann model, along with their contribution *d*_*m*_, size (in voxels) and expected ranking ER, across folds.

**Table 8:** MKL modelling of the comparison of ‘faces’ vs ‘houses’, based on the Brodmann atlas

Region	Area Description	Contribution (%)	Size	ER
brodmann area 19	Associative visual cortex (V3,V4,V5)	69.301764	589	70.00
brodmann area 30	Part of cingulate cortex	8.895519	179	68.25
brodmann area 23	Ventral posterior cingulate cortex	8.204297	117	65.13
brodmann area 37	Fusiform gyrus	5.712166	271	50.50
brodmann area 33	Part of anterior cingulate cortex	2.137109	10	65.25
Caudate Tail		1.619009	25	30.29
Midline Nucleus		1.272252	4	40.33
Hypothalamus		0.782331	10	34.91
brodmann area 27	Piriform cortex	0.778436	10	45.46
brodmann area 36	Part of the perirhinal cortex	0.251014	118	8.00
Lateral Dorsal Nucleus		0.236659	10	25.92
brodmann area 46	Dorsolateral prefrontal cortex	0.232491	91	8.00
Caudate Head		0.173804	120	5.42
Amygdala		0.083022	94	5.17
brodmann area 1	Part of primary somatosensory cortex	0.075739	43	5.21
brodmann area 29	Retrosplenial cingulate cortex	0.064846	37	2.67
Anterior Commissure		0.059758	5	5.42
Subthalamic Nucleus		0.059646	14	5.29
Medial Dorsal Nucleus		0.037006	64	2.67
Medial Geniculum Body		0.019957	6	2.54
Lateral Geniculum Body		0.003176	4	2.67

Regions ranked further (i.e. rank >21) have a perfectly null contribution to the model. ER stands for Expected Ranking. The region size is displayed in voxels. The weights were averaged across folds (except for computing ER)

### Regions Selected in MKL-HCP

Table 9 displays the regions with non-null contributions to the MKL-HCP model, along with their contribution *d*_*m*_, size (in voxels) and expected ranking ER, across folds.

**Table 9:** MKL modelling of the comparison of ‘faces’ vs ‘houses’, based on the HCP atlas

Region	Area Description	Contribution (%)	Size	ER
VMV1_ROI	VentroMedial Visual Area 1	52.05	143.00	180.00
IP0_ROI	Area IntraParietal 0	24.92	92.00	179.00
VMV2_ROI	VentroMedial Visual Area 2	10.26	54.00	170.50
7PL_ROI	Lateral Area 7P of the superior parietal cortex	4.91	44.00	169.00
MST_ROI	Medial Superior Temporal Area	3.66	28.00	153.96
IFJa_ROI	Area IFJa (part of the inferior frontal cortex)	3.31	54.00	161.08
DVT_ROI	Dorsal Transitional Visual Area	0.36	102.00	21.88
PHA2_ROI	ParaHippocampal Area 2	0.19	60.00	14.54
d23ab_ROI	Area dorsal 23 a + b (part of the posterior cingulate cortex)	0.16	71.00	14.46
V7_ROI	Seventh Visual Area	0.07	62.00	7.29
PGp_ROI	Area PGp (part of the Inferior Parietal Cortex)	0.05	109.00	14.46
Ig_ROI	Insular Granular Complex	0.05	61.00	21.67
52_ROI	Area 2 (part of insula and frontal operculum)	0.03	16.00	14.50

Regions ranked further (i.e. rank >13) have a perfectly null contribution to the model. ER stands for Expected Ranking. The region size is displayed in voxels. The weights were averaged across folds (except for computing ER)

## Face Data

### Regions Selected in MKL-AAL

Table 10 displays the regions with non-null contributions to the MKL-AAL model, along with their contribution *d*_*m*_, size (in voxels) and expected ranking ER, across folds.

**Table 10:** MKL modelling of the comparison of ‘faces’ vs ‘scrambled’, based on the AAL atlas

Region	Contribution (%)	Size	ER
Precentral_L	26.41	969.00	104.85
Cingulum_Mid_L	18.72	557.00	103.85
Frontal_Inf_Tri_R	9.96	474.00	98.12
Cuneus_L	5.71	433.00	91.77
Pallidum_L	5.18	71.00	98.88
Occipital_Inf_R	5.11	275.00	99.12
Heschl_R	4.94	67.00	95.31
Frontal_Inf_Oper_L	3.96	296.00	71.96
Caudate_R	3.79	243.00	86.85
Amygdala_L	3.39	66.00	96.77
Vermis_7	1.45	19.00	65.04
Occipital_Mid_R	1.42	560.00	51.00
Occipital_Mid_L	1.29	954.00	60.96
SupraMarginal_L	1.25	371.00	53.81
Frontal_Mid_Orb_R	1.09	186.00	64.46
Frontal_Sup_Orb_L	0.99	91.00	60.42
Angular_L	0.98	330.00	57.08
Caudate_L	0.65	228.00	14.85
Parietal_Inf_R	0.58	340.00	32.27
Frontal_Mid_Orb_L	0.55	214.00	18.12
Insula_R	0.54	510.00	4.00
Cerebelum_Crus1_L	0.54	73.00	21.92
Cingulum_Post_R	0.32	94.00	14.50
Occipital_Sup_R	0.31	386.00	17.85
Precuneus_L	0.17	931.00	10.58
Postcentral_R	0.14	855.00	3.65
Cingulum_Post_L	0.10	141.00	10.35
Postcentral_L	0.10	1060.00	3.62
Temporal_Sup_L	0.09	702.00	3.65
Vermis_9	0.06	7.00	7.04
Angular_R	0.06	469.00	3.62
Temporal_Mid_L	0.06	1354.00	3.58
Paracentral_Lobule_R	0.05	211.00	3.65
Lingual_R	0.05	674.00	3.58
Frontal_Inf_Oper_R	0.02	355.00	3.54
Occipital_Sup_L	0.00	374.00	3.42
Pallidum_R	0.00	75.00	3.31

Regions ranked further (i.e. rank >37) have a perfectly null contribution to the model. ER stands for Expected Ranking. The region size is displayed in voxels. The weights were averaged across folds (except for computing ER). A region label ending in ‘L’ (resp. ‘R’) means left (resp. right) hemisphere region

### Regions Selected in MKL-Brodmann

Table 11 displays the regions with non-null contributions to the MKL-Brodmann model, along with their contribution *d*_*m*_, size (in voxels) and expected ranking ER, across folds.

**Table 11:** MKL modelling of the comparison of ‘faces’ vs ‘scrambled’, based on the Brodmann atlas

Region	Area Description	Contribution (%)	Size	ER
brodmann area 31	Dorsal posterior cingulate cortex	21.92	469.00	68.46
brodmann area 44	Pars opercularis of the inferior frontal gyrus	16.41	102.00	65.38
brodmann area 5	Somatosensory Association Cortex	14.15	185.00	66.58
Substania Nigra		11.54	20.00	65.81
brodmann area 1	Part of primary somatosensory cortex	9.03	48.00	64.81
brodmann area 25	Subgenual area (part of the Ventromedial prefrontal cortex)	5.70	6.00	62.58
brodmann area 42	Auditory cortex	5.49	88.00	57.73
Caudate Tail		5.13	26.00	55.50
brodmann area 40	Supramarginal gyrus	2.03	900.00	42.08
brodmann area 7	Superior Parietal lobule and part of precuneus	1.64	1069.00	30.23
Hypothalamus		0.96	6.00	34.31
brodmann area 41	Auditory cortex	0.75	101.00	20.73
Caudate Head		0.70	88.00	29.08
Caudate Body		0.66	189.00	9.23
brodmann area 21	Middle temporal gyrus	0.55	493.00	13.65
brodmann area 46	Dorsolateral prefrontal cortex	0.51	190.00	13.81
brodmann area 45	Pars triangularis of the inferior frontal gyrus	0.48	174.00	11.50
Lateral Posterior Nucleus		0.48	9.00	29.31
brodmann area 6	Premotor cortex and Supplementary motor Cortex	0.47	1821.00	13.54
Lateral Globus Pallidus		0.41	121.00	4.92
Mammillary Body		0.38	22.00	9.12
Putamen		0.22	502.00	4.65
Ventral Posterior Medial Nucleus		0.10	8.00	6.58
brodmann area 2	Part of primary somatosensory cortex	0.09	203.00	2.19
brodmann area 34	Dorsal entorhinal cortex	0.06	51.00	2.23
brodmann area 11	Part of the orbitofrontal cortex	0.04	273.00	2.23
brodmann area 22	Superior temporal gyrus	0.03	469.00	2.23
brodmann area 39	Angular gyrus	0.03	290.00	4.42
brodmann area 24	Ventral anterior cingulate cortex	0.01	396.00	2.23
brodmann area 27	Piriform cortex	0.01	10.00	2.08
brodmann area 33	Part of anterior cingulate cortex	0.00	8.00	2.08
brodmann area 4	Primary motor cortex	0.00	339.00	2.19
Corpus Callosum		0.00	845.00	2.15

Regions ranked further (i.e. rank >33) have a perfectly null contribution to the model. ER stands for Expected Ranking. The region size is displayed in voxels. The weights were averaged across folds (except for computing ER)

### Regions Selected in MKL-HCP

Table 12 displays the regions with non-null contributions to the MKL-HCP model, along with their contribution *d*_*m*_, size (in voxels) and expected ranking ER, across folds.

**Table 12:** MKL modelling of the comparison of ‘faces’ vs ‘scrambled’, based on the HCP atlas

Region	Area Description	Contribution (%)	Size	ER
2_ROI	Area 2 (part of the somatosensory cortex)	18.58	319.00	179.92
PCV_ROI	PreCuneus Visual Area	8.04	119.00	176.62
6d_ROI	Dorsal area 6 (superior premotor subdivions)	7.61	123.00	175.77
V6_ROI	Sixth Visual Area	7.04	101.00	155.77
PFcm_ROI	Area PFcm (part of the auditory cortex)	6.74	115.00	175.00
TE1m_ROI	Area TE1 Middle (lateral temporal cortex)	5.41	98.00	147.54
11l_ROI	Area 11 l (part of the orbital and polar frontal cortex)	5.34	132.00	172.81
44_ROI	Area 44 (part of the inferior frontal cortex)	4.58	197.00	130.65
AAIC_ROI	Anterior Agranular Insula Complex	3.87	123.00	138.88
31a_ROI	Area 31a (part of the posterior cingulate cortex)	3.46	68.00	54.54
TPOJ3_ROI	Area TemporoParietoOccipital Junction 3	3.16	46.00	149.81
PeEc_ROI	Perirhinal Ectorhinal Cortex	2.59	202.00	142.96
7AL_ROI	Lateral area 7A of the superior parietal cortex	2.23	102.00	110.92
MI_ROI	Middle Insular Area	2.06	155.00	97.65
10pp_ROI	Polar 10p (part of orbital and polar frontal cortex)	1.66	9.00	115.23
8BM_ROI	Area 8BM (medial prefrontal cortex)	1.56	231.00	96.27
5mv_ROI	Area 5 m ventral of the superior parietal cortex	1.42	107.00	114.77
6v_ROI	Ventral Area 6 (inferior premotor subdivisions)	1.22	131.00	107.58
MBelt_ROI	Medial Belt Complex (Early Auditory Cortex)	1.14	67.00	113.42
PoI2_ROI	Posterior Insular Area 2	1.00	174.00	69.96
A5_ROI	Auditory 5 Complex (Auditory Association Cortex)	0.93	212.00	33.08
A4_ROI	Auditory 4 Complex	0.87	120.00	87.62
24dd_ROI	Dorsal Area 24d (cingulate motor areas)	0.77	160.00	38.73
10d_ROI	Area 10d (part of Orbital and Polar Frontal Cortex)	0.67	88.00	44.88
PGs_ROI	Area PGs (part of inferior parietal cortex)	0.65	227.00	68.88
VMV2_ROI	VentroMedial Visual Area	0.59	49.00	75.65
PFm_ROI	Area PFm Complex (part of Inferior parietal cortex)	0.57	312.00	74.54
pOFC_ROI	posterior OFC Complex (orbital frontal cortex)	0.54	19.00	74.92
AVI_ROI	Anterior Ventral Insular Area	0.52	116.00	25.88
PoI1_ROI	Area Posterior Insular 1	0.45	108.00	32.12
s6-8_ROI	Superior 6–8 Transitional Area (part of dorsolateral prefrontal cortex)	0.42	107.00	37.85
IP2_ROI	Area IntraParietal 2	0.40	56.00	25.85
IFJa_ROI	Area IFJa (part of the inferior frontal cortex)	0.36	59.00	37.62
FST_ROI	Area FST (Visual Area)	0.32	70.00	56.12
31pv_ROI	Area 31p ventral (part of the posterior cingulate cortex)	0.30	84.00	73.96
25_ROI	Area 25 (part of Anterior Cingulate and Medial Prefrontal Cortex)	0.26	8.00	25.31
PHT_ROI	Area PHT (part of lateral temporal cortex)	0.25	168.00	13.15
55b_ROI	Area 55b (part of premotor cortex)	0.23	115.00	19.46
OP1_ROI	Area OP1/SII (Part of Posterior Opercular Cortex)	0.22	92.00	12.73
8BL_ROI	Area 8B Lateral (Part of dorsolateral prefrontal cortex)	0.20	223.00	6.65
47s_ROI	Area 47 s (part of orbital and polar frontal cortex)	0.20	128.00	19.19
23d_ROI	Area 23d (part of posterior cingulate cortex)	0.13	100.00	31.15
V2_ROI	Second Visual Area	0.13	744.00	12.62
47m_ROI	Area 47 m (part of orbital and polar frontal cortex)	0.12	70.00	24.88
IP1_ROI	Area IntraParietal 1	0.12	148.00	6.54
3b_ROI	Primary somatosensory cortex	0.11	268.00	6.38
V3B_ROI	Area V3b (part of dorsal stream visual cortex)	0.11	20.00	36.58
6ma_ROI	Area 6 m anterior (part of supplementary motor cortex)	0.11	235.00	12.62
45_ROI	Area 45 (part of inferior frontal cortex)	0.11	159.00	24.46
8Av_ROI	Area 8Av (part of dorsolateral prefrontal cortex)	0.09	224.00	12.65
7m_ROI	Area 7 m (part of posterior cingulate cortex)	0.08	122.00	6.54
46_ROI	Area 46 (part of dorsolateral prefrontal cortex)	0.07	172.00	19.15
PGp_ROI	Area PGp (part of the Inferior Parietal Cortex)	0.06	144.00	6.35
FEF_ROI	Frontal Eye Fields	0.05	106.00	6.31
IP0_ROI	Area IntraParietal 0	0.04	89.00	6.35
RI_ROI	RetroInsular Cortex	0.04	64.00	6.12
7Pm_ROI	Medial Area 7P (part of the superior medial parietal cortex)	0.03	81.00	12.58
OFC_ROI	Orbitofrontal cortex	0.03	14.00	6.15
4_ROI	Primary Motor Cortex	0.03	477.00	12.27
PIT_ROI	Posterior InferoTemporal	0.02	71.00	6.23
a47r_ROI	Area anterior 47r (part of inferior frontal cortex)	0.01	276.00	18.04
LBelt_ROI	Lateral Belt Complex (early auditory areas)	0.01	43.00	6.00
V8_ROI	Eighth Visual Area	0.01	94.00	6.08
A1_ROI	Primary Auditory Cortex	0.01	51.00	12.12
d23ab_ROI	Area dorsal 23 a + b (part of the posterior cingulate cortex)	0.01	75.00	6.04
V3A_ROI	Area V3A (dorsal stream areas)	0.01	117.00	6.27

Regions ranked further (i.e. rank >66) have a perfectly null contribution to the model. ER stands for Expected Ranking. The region size is displayed in voxels. The weights were averaged across folds (except for computing ER)

## OASIS Data

### Regions Selected in MKL-AAL

Table 13 displays the regions with non-null contributions to the MKL-AAL model, along with their contribution *d*_*m*_, size (in voxels) and expected ranking ER, across folds.

**Table 13:** MKL modelling of the comparison of ‘demented’ vs ‘healthy’, based on the AAL atlas

Region	Contribution (%)	Size	ER
Frontal_Inf_Tri_L	12.39	3507.00	115.20
Lingual_L	7.59	4338.00	111.80
Hippocampus_R	6.32	1353.00	88.90
Frontal_Inf_Oper_R	5.92	2049.00	88.80
Thalamus_L	5.14	1113.00	108.30
Temporal_Inf_L	4.63	6361.00	97.80
Frontal_Sup_L	4.47	3910.00	56.20
Cerebelum_Crus1_L	4.23	4519.00	87.40
Insula_L	4.02	3528.00	86.90
Cingulum_Mid_R	3.45	4483.00	64.10
Fusiform_R	3.23	5351.00	65.00
Precuneus_L	3.21	5505.00	101.30
Temporal_Mid_L	2.70	9111.00	83.20
Temporal_Pole_Mid_R	2.61	1657.00	62.70
Precentral_R	1.96	3371.00	43.00
SupraMarginal_L	1.75	2075.00	42.10
Hippocampus_L	1.75	1406.00	61.00
Cingulum_Ant_L	1.64	2969.00	52.30
Frontal_Sup_R	1.47	4555.00	52.50
Cerebelum_Crus1_R	1.34	4276.00	50.00
Frontal_Sup_Medial_R	1.31	2694.00	41.10
Precentral_L	1.25	3761.00	59.70
Heschl_R	1.12	517.00	41.70
Calcarine_L	0.96	4519.00	40.50
Angular_L	0.91	2023.00	38.80
Vermis_10	0.89	29.00	77.80
Frontal_Mid_L	0.80	6488.00	30.50
Parietal_Inf_R	0.77	2104.00	75.40
Vermis_7	0.73	393.00	65.40
Frontal_Inf_Orb_R	0.69	3061.00	39.40
Putamen_L	0.65	1711.00	66.50
Occipital_Mid_R	0.65	3564.00	47.10
Cuneus_L	0.63	2601.00	37.70
Frontal_Inf_Tri_R	0.62	2777.00	20.60
Cerebelum_Crus2_R	0.60	3309.00	39.50
Frontal_Sup_Orb_R	0.51	1618.00	30.60
Supp_Motor_Area_L	0.48	3116.00	20.10
Cerebelum_3_L	0.45	159.00	36.20
Cuneus_R	0.43	2475.00	37.60
Cerebelum_10_L	0.42	6.00	37.10
Supp_Motor_Area_R	0.35	3347.00	28.20
Occipital_Inf_L	0.35	1872.00	10.60
Cerebelum_4_5_R	0.33	1818.00	36.60
Frontal_Inf_Oper_L	0.32	1524.00	37.10
Temporal_Pole_Sup_R	0.30	1412.00	26.90
Occipital_Mid_L	0.28	5641.00	10.30
Postcentral_R	0.27	3979.00	10.50
Temporal_Pole_Mid_L	0.27	1175.00	27.80
Pallidum_L	0.27	229.00	28.10
Cerebelum_7b_L	0.26	808.00	19.40
Caudate_R	0.23	1111.00	36.60
Occipital_Sup_L	0.23	1710.00	10.00
Cerebelum_7b_R	0.22	705.00	18.40
Vermis_9	0.19	321.00	27.20
Frontal_Inf_Orb_L	0.18	3303.00	10.10
Lingual_R	0.17	4625.00	9.90
Precuneus_R	0.14	5142.00	17.70
SupraMarginal_R	0.12	3395.00	9.70
Parietal_Sup_L	0.08	2274.00	9.10
Cingulum_Ant_R	0.07	2460.00	9.10
Caudate_L	0.07	988.00	9.00
Heschl_L	0.07	485.00	9.00
Temporal_Pole_Sup_L	0.06	1440.00	8.90
ParaHippocampal_L	0.06	1964.00	16.90
Cerebelum_8_L	0.06	1902.00	17.40
Cingulum_Post_L	0.05	699.00	19.20
Cerebelum_10_R	0.05	8.00	16.80
Angular_R	0.05	2871.00	8.90
Cerebelum_Crus2_L	0.05	3514.00	8.70
Thalamus_R	0.05	1178.00	9.20
Cingulum_Post_R	0.03	369.00	8.50
Vermis_1_2	0.02	79.00	8.90
Frontal_Mid_Orb_R	0.02	1798.00	18.10

Regions ranked further (i.e. rank >73) have a perfectly null contribution to the model. ER stands for Expected Ranking. The region size is displayed in voxels. The weights were averaged across folds (except for computing ER). A region label ending in ‘L’ (resp. ‘R’) means left (resp. right) hemisphere region

### Regions Selected in MKL-Brodmann

Table 14 displays the regions with non-null contributions to the MKL-Brodmann model, along with their contribution *d*_*m*_, size (in voxels) and expected ranking ER, across folds.

**Table 14:** MKL modelling of the comparison of ‘demented’ vs ‘healthy’, based on the Brodmann atlas

Region	Area Description	Contribution (%)	Size	ER
brodmann area 7	Superior Parietal lobule and part of precuneus	11.56	6488.00	63.10
brodmann area 46	Dorsolateral prefrontal cortex	9.16	740.00	62.50
brodmann area 6	Premotor cortex and Supplementary motor Cortex	8.80	8215.00	61.90
brodmann area 22	Superior temporal gyrus	7.26	2439.00	58.00
brodmann area 44	Pars opercularis of the inferior frontal gyrus	6.51	885.00	59.30
brodmann area 45	Pars triangularis of the inferior frontal gyrus	5.58	546.00	56.50
brodmann area 18	Secondary visual cortex (V2)	4.36	4810.00	49.30
brodmann area 24	Ventral anterior cingulate cortex	4.07	2345.00	54.40
brodmann area 20	Inferior temporal gyrus	3.89	4218.00	50.80
brodmann area 17	Primary visual cortex (V1)	3.47	1101.00	53.70
Caudate Tail		3.36	8.00	54.40
brodmann area 19	Associative visual cortex (V3,V4,V5)	2.94	4725.00	43.90
brodmann area 38	Temporopolar area	2.73	2471.00	42.70
brodmann area 34	Dorsal entorhinal cortex	2.40	608.00	36.90
brodmann area 39	Angular gyrus	1.87	1783.00	39.10
brodmann area 3	Part of primary somatosensory cortex	1.76	1064.00	26.70
brodmann area 2	Part of primary somatosensory cortex	1.68	812.00	39.20
brodmann area 10	Anterior prefrontal cortex	1.54	4066.00	30.30
brodmann area 42	Auditory cortex	1.48	433.00	38.90
brodmann area 23	Ventral posterior cingulate cortex	1.47	866.00	26.00
brodmann area 36	Ectorhinal area	1.39	1032.00	33.40
Caudate Body		1.28	521.00	37.70
Hippocampus		1.28	572.00	21.00
brodmann area 47	Pars orbitalis, part of the inferior frontal gyrus	1.18	2550.00	20.80
Red Nucleus		1.17	7.00	36.70
brodmann area 1	Part of primary somatosensory cortex	1.01	57.00	31.90
Putamen		0.92	2452.00	28.90
Mammillary Body		0.90	60.00	31.60
Lateral Posterior Nucleus		0.59	45.00	26.60
brodmann area 8	Part of the frontal cortex, it includes the frontal eye fields	0.57	2473.00	14.50
Ventral Lateral Nucleus		0.51	74.00	25.50
brodmann area 5	Somatosensory Association Cortex	0.49	1044.00	18.70
brodmann area 31	Dorsal Posterior cingulate cortex	0.48	3310.00	13.60
brodmann area 40	Supramarginal gyrus	0.39	4753.00	9.50
Lateral Globus Pallidus		0.28	53.00	12.70
brodmann area 9	Dorsolateral prefrontal cortex	0.27	3740.00	12.80
brodmann area 29	Retrosplenial cingulate cortex	0.27	203.00	13.10
Medial Dorsal Nucleus		0.26	573.00	13.10
Medial Geniculum Body		0.22	5.00	20.80
brodmann area 11	Part of the orbitofrontal cortex	0.20	4067.00	5.40
brodmann area 13	Insular Cortex	0.18	3527.00	8.20
brodmann area 33	Part of anterior cingulate cortex	0.16	26.00	20.00
Corpus Callosum		0.08	109.00	4.10
Caudate Head		0.03	740.00	4.40
Ventral Posterior Medial Nucleus		0.00	49.00	3.70
brodmann area 28	Ventral entorhinal cortex	0.00	609.00	3.80

Regions ranked further (i.e. rank >46) have a perfectly null contribution to the model. ER stands for Expected Ranking. The region size is displayed in voxels. The weights were averaged across folds (except for computing ER)

### Regions Selected in MKL-HCP

Table 15 displays the regions with non-null contributions to the MKL-HCP model, along with their contribution *d*_*m*_, size (in voxels) and expected ranking ER, across folds.

**Table 15:** MKL modelling of the comparison of ‘demented’ vs ‘healthy’, based on the HCP atlas

Region	Area Description	Contribution (%)	Size	ER
H_ROI	Hippocampus	10.53	1050.00	177.90
23c_ROI	Area 23c (part of posterior cingulate cortex)	7.30	1000.00	175.60
TE2a_ROI	Area TE2 anterior (part of lateral temporal cortex)	5.26	2039.00	152.50
6d_ROI	Dorsal area 6 (superior premotor subdivions)	5.23	563.00	156.50
IP2_ROI	Area IntraParietal 2	4.48	441.00	172.20
V1_ROI	Primary Visual Cortex	4.31	5459.00	154.60
STSdp_ROI	Area STSd posterior (auditory association cortex)	3.61	926.00	166.10
31a_ROI	Area 31a (part of the posterior cingulate cortex)	3.44	588.00	120.40
9-46d_ROI	Area 9-46d (part of the cortex prefrontal dorsolateral)	3.08	1362.00	135.40
7Am_ROI	Medial Area 7A (Superior Parietal Cortex)	3.00	852.00	151.00
24dv_ROI	Ventral Area 24d (Cingulate motor area)	2.89	417.00	135.20
6a_ROI	Area 6 anterior (premotor subdivisions)	2.80	1313.00	127.80
47l_ROI	Area 47 l (47 lateral) (part of inferior frontal gyrus)	2.44	810.00	117.30
AAIC_ROI	Anterior Agranular Insula Complex	2.41	831.00	100.40
V4_ROI	Fourth Visual Area	2.31	1692.00	132.20
STV_ROI	Superior Temporal Visual Area	1.89	1057.00	82.90
8C_ROI	Area 8C (part of inferior frontal cortex)	1.88	1018.00	113.90
PFop_ROI	Area PF opercular (part of inferior parietal cortex)	1.64	753.00	66.90
IFSp_ROI	Area IFSp (part of inferior frontal sulcus)	1.38	578.00	66.70
44_ROI	Area 44 (part of the inferior frontal cortex)	1.30	1142.00	97.00
6ma_ROI	Area 6 m anterior (Part of supplementary motor cortex)	1.24	1103.00	97.50
AVI_ROI	Anterior Ventral Insular Area	1.16	881.00	97.30
TGv_ROI	Area TG Ventral (part of lateral temporal cortex)	1.13	1772.00	50.30
PIT_ROI	Posterior InferoTemporal	1.10	437.00	111.00
p10p_ROI	Area posterior 10p (Part of orbital and polar frontal cortex)	1.06	776.00	80.60
IP0_ROI	Area IntraParietal 0	0.99	711.00	65.80
V3_ROI	Third Visual Area	0.95	2777.00	80.50
31pv_ROI	Area 31p ventral (Part of the posterior cingulate cortex	0.94	685.00	94.90
p9-46v_ROI	Area posterior 9-46v (part of dorsolateral prefrontal cortex)	0.94	992.00	50.30
a24pr_ROI	Anterior 24 prime (part of anterior cingulate)	0.92	667.00	94.60
TGd_ROI	Area TG dorsal (part of lateral temporal cortex)	0.87	5074.00	47.90
p24_ROI	Area posterior 24 (part of anterior cingulate)	0.85	867.00	65.60
8BL_ROI	Area 8B Lateral (part of dorsolateral prefrontal cortex)	0.81	674.00	64.10
7Pm_ROI	Medial Area 7P (part of the superior medial parietal cortex)	0.78	611.00	17.80
7PL_ROI	Lateral Area 7P of the superior parietal cortex	0.75	284.00	49.50
V2_ROI	Second Visual Area	0.71	5166.00	17.70
TPOJ1_ROI	Area TemporoParietoOccipital Junction 1	0.67	1069.00	48.90
V3B_ROI	Area V3b (part of dorsal stream visual cortex)	0.66	139.00	94.40
A4_ROI	Auditory 4 Complex	0.64	638.00	78.80
p24pr_ROI	Area Posterior 24 prime (part of anterior cingulate)	0.60	809.00	93.20
1_ROI	Area 1 (part of primary somatosensory complex)	0.58	341.00	17.70
TE2p_ROI	Area TE2 posterior (part of lateral temporal cortex)	0.57	1526.00	32.90
V7_ROI	Seventh Visual Area	0.54	391.00	78.70
LIPd_ROI	Area Lateral IntraParietal dorsal (Part of superior parietal cortex)	0.49	208.00	61.90
MST_ROI	Medial Superior Temporal Area	0.47	230.00	47.80
FOP1_ROI	Frontal Opercular area 1 (Part of posterior opercular cortex)	0.46	565.00	48.60
45_ROI	Area 45 (part of inferior frontal gyrus)	0.44	944.00	17.40
IP1_ROI	Area IntraParietal 1	0.42	1044.00	32.20
10v_ROI	Area 10v (part of medial prefrontal cortex)	0.41	1569.00	62.20
33pr_ROI	Area 33 prime (part of anterior cingulate cortex)	0.40	305.00	76.30
6mp_ROI	Area 6mp (supplementary motor area)	0.39	1081.00	17.30
8BM_ROI	Area 8BM (medial prefrontal cortex)	0.37	1828.00	75.90
5mv_ROI	Area 5 m ventral of the superior parietal cortex	0.37	600.00	16.90
PH_ROI	Area PH (lies between the MT+ complex and the ventral stream)	0.36	1473.00	47.20
s32_ROI	Area s32 (part of anterior cingulate and medial prefrontal cortex)	0.36	500.00	30.80
TF_ROI	Area TF (part of lateral temporal cortex)	0.35	2309.00	31.30
VMV1_ROI	VentroMedial Visual Area 1	0.35	1189.00	61.40
55b_ROI	Area 55b (part of premotor cortex)	0.34	658.00	62.10
7AL_ROI	Lateral area 7A of the superior parietal cortex	0.34	612.00	62.90
11l_ROI	Area 11 l (part of the orbital and polar frontal cortex)	0.33	1458.00	46.30
V6_ROI	Sixth Visual Area	0.28	588.00	61.30
SFL_ROI	Superior Frontal Language Area	0.26	839.00	31.00
POS2_ROI	Parieto-Occipital Sulcus Area 2 (part of posterior cingulate cortex)	0.25	1761.00	45.50
PeEc_ROI	Perirhinal Ectorhinal Cortex	0.24	2622.00	16.50
TE1p_ROI	Area TE1 posterior (part of lateral temporal cortex	0.23	2090.00	16.60
PHA2_ROI	ParaHippocampal Area 2	0.23	523.00	45.70
MIP_ROI	Medial IntraParietal Area	0.19	482.00	16.20
p47r_ROI	Area posterior 47r (Part of inferior frontal cortex)	0.19	718.00	16.20
3a_ROI	Area 3a (Part of Primary somatosensory cortex)	0.17	190.00	44.90
RI_ROI	RetroInsular Cortex	0.11	360.00	15.70
6r_ROI	Rostral Area 6 (Part of inferior premotor subdivisions	0.11	1434.00	31.50
PFcm_ROI	Area PFcm (part of the auditory cortex)	0.08	846.00	29.60
FST_ROI	Area FST (Visual Area)	0.08	584.00	15.20
5m_ROI	Area 5 m (part of paracentral lobule)	0.07	603.00	30.40
AIP_ROI	Anterior IntraParietal Area	0.06	813.00	14.70
10pp_ROI	Polar 10p (part of orbital and polar frontal cortex)	0.05	789.00	14.60
8Av_ROI	Area 8Av (part of dorsolateral prefrontal cortex)	0.03	1029.00	15.40
TPOJ2_ROI	Area TemporoParietoOccipital Junction 2	0.02	956.00	14.10
pOFC_ROI	posterior OFC Complex (orbital frontal cortex	0.02	1096.00	14.70
V4t_ROI	Area V4 t (part of MT+ Complex and Neighboring Visual Areas)	0.01	235.00	14.60
a9-46v_ROI	Area anterior 9-46v (part of dorsolateral prefrontal cortex)	0.00	899.00	15.00
OP4_ROI	Area OP4/PV (part of opercular cortex)	0.00	1128.00	15.20
PSL_ROI	PeriSylvian Language Area	0.00	854.00	14.40
25_ROI	Area 25 (part of Anterior Cingulate and Medial Prefrontal Cortex)	0.00	606.00	13.60
EC_ROI	Entorhinal Cortex	0.00	1008.00	15.30

Regions ranked further (i.e. rank >85) have a perfectly null contribution to the model. ER stands for Expected Ranking. The region size is displayed in voxels. The weights were averaged across folds (except for computing ER)

## Footnotes

1 Please note that this introduction discusses the most used approaches, but does not represent an exhaustive list.

2 Note that by sparse machine learning models we mean models that are sparse in the feature space (e.g. LASSO, Elastic Net) and not models that are sparse in the kernel space (e.g. Support Vector Machine).

3 As this is a single subject data set, co-registration, normalization and smoothing are not a necessary step.
